# DNA methylation and whole-genome transcription analysis in CD4^+^ T cells from systemic lupus erythematosus patients with or without renal damage

**DOI:** 10.1186/s13148-024-01699-7

**Published:** 2024-07-30

**Authors:** Xiaomin Liu, Siyu Zhou, Mengjie Huang, Ming Zhao, Weiguang Zhang, Qun Liu, Kangkang Song, Xu Wang, Jiaona Liu, Qing OuYang, Zheyi Dong, Ming Yang, Zhenzhen Li, Li Lin, Yi Liu, Yang Yu, Simin Liao, Jian Zhu, Lin Liu, Wenge Li, Linpei Jia, Aihua Zhang, Chaomin Guo, LiuYang Yang, Qing gang Li, Xueyuan Bai, Ping Li, Guangyan Cai, Qianjin Lu, Xiangmei Chen

**Affiliations:** 1 Department of Nephrology, The First Medical Center, Chinese PLA General Hospital, Chinese PLA Institute of Nephrology, State Key Laboratory of Kidney Diseases, National Clinical Research Center for Kidney Diseases, General Hospital of People’s Liberation Army (301 Hospital), Haihe Laboratory of Cell Ecosystem, 28 Fuxing Road Beijing (wukesong), Beijing, 100853 China; 2 Hunan Key Laboratory of Medical Epigenomics, Department of Dermatology, The Second Xiangya Hospital of Central South University, Changsha, China; 3 Department of Nephrology, Beijing Tongren Hospital, Capital Medical University, Beijing, China; 4 Department of Blood Transfusion, The First Medical Center, Chinese PLA General Hospital, Beijing, China; 5 Department of Rheumatology and Immunology, The First Medical Center, Chinese PLA General Hospital, Beijing, China; 6 Department of Nephrology, China-Japan Friendship Hospital, Beijing, China; 7 Department of Nephrology, Xuanwu Hospital, Capital Medical University, Beijing, China; 8 Laboratory Medicine Department, First Medical Center of Chinese PLA General Hospital, Beijing, China; 9 Key Laboratory of Basic and Translational Research On Immune-Mediated Skin Diseases, Chinese Academy of Medical Sciences, Jiangsu Key Laboratory of Molecular Biology for Skin Diseases and STIs, Institute of Dermatology, Chinese Academy of Medical Sciences and Peking Union Medical College, #12 Jiangwangmiao Street, Nanjing, 210042 China

**Keywords:** LN, CD4^+^ T cell, BCL2L14, DNA methylation

## Abstract

**Background:**

Lupus nephritis (LN) is the most common cause of kidney injury in systemic lupus erythematosus (SLE) patients and is associated with increased mortality. DNA methylation, one of the most important epigenetic modifications, has been reported as a key player in the pathogenesis of SLE. Hence, our article aimed to explore DNA methylation in CD4^+^ T cells from LNs to identify additional potential biomarkers and pathogenic genes involved in the progression of LN.

**Methods:**

Our study enrolled 46 SLE patients with or without kidney injury and 23 healthy controls from 2019 to 2022. CD4^+^ T cells were sorted for DNA methylation genotyping and RNA-seq. Through bioinformatics analysis, we identified the significant differentially methylated CpG positions (DMPs) only in the LN group and validated them by Bisulfite PCR. Integration analysis was used to screen for differentially methylated and expressed genes that might be involved in the progression of LN, and the results were analyzed via cell experiments and flow cytometry.

**Results:**

We identified 243 hypomethylated sites and 778 hypermethylated sites only in the LN cohort. Three of these DMPs, cg08332381, cg03297029, and cg16797344, were validated by Bisulfite PCR and could be potential biomarkers for LN. Integrated analysis revealed that the expression of *BCL2L14* and *IFI27* was regulated by DNA methylation, which was validated by azacytidine (5-aza) treatment. The overexpression of *BCL2L14* in CD4^+^ T cells might induce renal fibrosis and inflammation by regulating the differentiation and function of Tfh cells.

**Conclusion:**

Our study identified novel aberrant DMPs in CD4^+^ T cells only in LN patients and DNA methylation-regulated genes that could be potential LN biomarkers. *BCL2L14* is likely involved in the progression of LN and might be a treatment target.

**Supplementary Information:**

The online version contains supplementary material available at 10.1186/s13148-024-01699-7.

## Introduction

Lupus nephritis (LN) is a systemic lupus erythematosus (SLE) kidney disease that frequently leads to end-stage kidney disease despite treatment [1, 2] and is often associated with poor prognosis. According to the cohorts reported in the literature, 20–75% of SLE patients, mainly prevalent in the Asia Pacific region, have LN [3–5]. LN typically develops early in the disease course, generally within the first 6 to 36 months, and death directly attributable to kidney disease occurs in 5% to 25% of patients with proliferative LNs within 5 years of onset [6]. The LN diagnosis relies on kidney biopsy, which might be limited by potential bleeding risk, medical level, and physical condition.

Many scholars believe that the heterogeneity of SLE results from interactions between genes and the environment. The presence of environmental factor-induced epigenetic modifications on immune cells may provide some insights [7]. Epigenetic modifications, which include DNA methylation, histone modifications, and noncoding RNA regulation, refer to heritable changes in a chromosome without altering the DNA sequence [8]. Increasing evidence has shown the importance of dysregulated epigenetic modifications in immune cells in the pathogenesis of lupus and has identified epigenetic changes as potential biomarkers and therapeutic targets [7].

However, far fewer DNA methylation studies have been aimed at treating LN than SLE. Several previous studies [9–11] have also performed DNA methylation screening in CD4^+^T cells or peripheral blood mononuclear cells (PBMCs) from SLE patients and found several DMPs or genes unique to the LN population, such as cg10152499 (*CHST12*) and cg15065340 (*TNK2)* gene [9, 10]. Only clinical diagnosis of LN and lack of pathology results allowed confounding factors that might limit the number and precision of differentially methylated genes unique to the LN group. Thus, in this article, we analyzed the DNA methylation distribution and variation in genome-wide and mRNA transcription profiles in CD4^+^ T cells from proliferative LN patients and SLE patients without kidney injury compared to healthy controls to identify potential biomarkers for LN. Integrated analysis of DNA methylation and mRNA expression also revealed that several genes might take part in the pathophysiology of LN, which was also initially explored.

## Materials and methods

### Sample collection

A total of 46 patients with SLE and 23 healthy individuals were enrolled between April 2020 and April 2022 at the Chinese PLA General Hospital and the Second Xiangya Hospital. The inclusion and exclusion criteria are listed in Table 1. The SLE patients were divided into two groups: the proliferative LN group and the SLE without kidney injury (referred to simply as the SLE-NKI) group. In the discovery phase, 24 samples (LN, SLE-NKI, and HC groups, 8 samples in each group) were included for Illumina Infinium Methylation EPIC Bead Chip (850 K) genotype and RNA-seq. Another 45 samples (LN, SLE-NKI, and HC groups, 15 samples in each group) were subjected to bisulfite sequencing PCR (BSP) and RT-qPCR for external validation.

**Table 1:** Inclusion and exclusion criteria

Group	Inclusion criteria	Exclusion criteria
Lupus nephritis	1. Age ≥ 18, female, Chinese Han population 2. Diagnosed as lupus nephritis [13], and renal biopsy results were IV or IV + V [14]	1. Complications include severe infection, other systemic disease, and malignancy 2. Mixed connective tissue disease, overlap syndrome 3. Smoke 4. Pregnancy 5. The biopsy result is lupus nephritis combined with another kind of kidney disease 6. Blood transfusion or dialysis
SLE without renal damage	1. Age ≥ 18, female, Chinese Han population 2. Diagnosed as SLE [12] 3. Without any sign of renal damage and renal damage history	1. Complications include severe infection, other systemic disease, and malignancy 2. Mixed connective tissue disease, overlap syndrome 3. Smoke 4. Pregnancy 5. Blood transfusion or dialysis
Health control	1. Age ≥ 18, female, Chinese Han population 2. Clinically assessed as a healthy population	1. Complications include severe infection, other systemic disease, and malignancy 2. Mixed connective tissue disease, overlap syndrome 3. Smoke 4. Pregnancy 5. Family history of SLE or other systemic disease

All patients in this study fulfilled at least 4 of the SLE classification criteria of the American College of Rheumatology [12]. Lupus nephritis was defined based on clinical and laboratory manifestations that met ACR criteria (persistent proteinuria > 0.5 g per day or more significant than 3+ according to dipstick tests and/or cellular casts, including red blood cells [13], hemoglobin, granular, tubular, or mixed) [14]. Furthermore, renal biopsy results were IV or IV + V. SLE-NKI is defined as SLE patients without any sign of renal damage. Healthy controls were recruited from the blood donation center at Chinese PLA General Hospital. All enrolled individuals in the three groups were matched for age and sex.

This study protocol was approved by the Medicine Ethics Committee of the Chinese People’s Liberation Army General Hospital (Approval No. S2019-095-01) and Second Xiangya Hospital (No. 201930044). All enrolled individuals provided written informed consents in accordance with the Declaration of Helsinki before initiation of the study.

### Cell isolation and DNA/RNA isolation

A total of 20 ml of peripheral venous blood was drawn from each patient and control subject and preserved with heparin ethylenediaminetetraacetic acid. PBMCs were isolated by Ficoll (GE Healthcare). Total CD4^+^ T cells were isolated by positive selection using CD4 beads, according to protocols provided by the manufacturer (Miltenyi Biotec, Germany). Naïve CD4^+^ T cells were isolated by negative selection using naïve CD4^+^ T cell isolation kit II (Miltenyi Biotec, Germany). The purities of the CD4^+^ T cells and naïve CD4^+^ T cells (CD4+ CD45RA+) were greater than 95%.

Total DNA was extracted from CD4^+^ T cells by a Quick-DNA™ MiniPrep (Zymo, D3024). Total RNA was isolated from each sample with TRIzol reagent (Thermo Fisher). The purity and amount of total RNA samples were determined with a NanoDrop ND-1000 (Thermo Fisher, Shanghai, China) according to the manufacturer’s instructions.

### DNA methylation genotyping and transcriptome sequencing

The Illumina Infinium Methylation EPIC BeadChip (850 K) was used for quantitatively investigating methylation sites across the genome at a single-nucleotide resolution according to the standard operational approach; this tool can quantitatively cover more than 850,000 methylation sites. We excluded probe sites with detection *p* value > 0.01, a bead count < 3 in ≥ 5% of the sample, and missing in ≥ 1% of the sample; additionally, we excluded sites predicted to hybridize to multiple loci. The methylation score for each CpG was calculated as a β value according to the fluorescence intensity ratio. A β value may take any value between 0 (nonmethylated) and 1 (completely methylated). The β values were extracted, filtered, and normalized using BMIQ (beta mixed integer-quantile normalization) [15]. The normalized methylation data were analyzed by significance analysis of microarray data (SAM, Stanford University) [26]. The significant differentially methylated CpG positions (DMPs) were calculated using the R package ‘Champ.DMP,’ and DMP with FDR < 0.05, and Δ β > 0.1 were selected [16].

Transcriptome sequencing was performed by NovaSeq 600 (Illumina). We used HTSeq(0.9.1) statistics [17] to compare the Read Count values on each gene as the original expression of the gene and then used FPKM [18] to standardize the expression. Then, difference expression of genes was analyzed by DESeq (1.30.0) with screened conditions as follows: expression difference multiple |log2FoldChange|> 1, significant P value < 0.05. At the same time, we used the R language Pheatmap (1.0.8) software package to perform bidirectional clustering analysis of all different genes of samples. We got a heatmap according to the expression level of the same gene in different samples and the expression patterns of different genes in the same sample with the Euclidean method to calculate the distance and the Complete Linkage method to cluster.

### Gene ontology and pathway analysis

Gene Ontology (GO) analysis was applied to determine the attributes of the gene products generated in terms of biological processes, cellular components, and molecular functions [19]. The Kyoto Encyclopedia of Genes and Genomes (KEGG) database was searched to determine the biochemical pathways enriched by these mRNAs [20]. GO and KEGG analyses were performed in Metascape [21] (http://metascape.org). The terms with *p* < 0.05 were dimed as significant. The enrichment score was calculated as − Log (*p* value).

### Bisulfite PCR (BS-PCR)

We selected 10 DMPs only in the LNs detected by BS-PCR in a large external cohort of 45 samples (15 for each group) to obtain potential biomarkers for LN, of which the│∆β│ > 0.2, *P* values between LN versus HC and LN versus SLE-NKI were < 0.05. BS-PCR based on the treatment of DNA with bisulfite can convert cytosine residues to uracil but does not affect 5-methylcytosine residues [22]. BS-PCR could show the context of methylation and determine absolute DNA methylation levels [22]. One milligram of genomic DNA was bisulfite-converted using the EZ DNA Methylation-Lighting™ Kit (Zymo Research, D5031). The DNA fragment was PCR amplified and cloned into the PMD 18-T vector (Takara, 6011). Ten independent clones from each subject were sequenced. The primer sequences and annealing temperatures used in the BS-PCR are shown in Additional file 1: S-Table 1.

### In vitro human CD4^+^ T cell activation

Total CD4^+^ T cells were isolated from the PBMCs of healthy donors and then cultured in complete GT-T551 medium (Takara) containing 10% fetal bovine serum (HyClone) under the stimulation of plate-bound anti-CD3(2 μg/mL, Millipore) and soluble anti-CD28(1 μg/mL, Millipore) for 24 h for follow-up experiments.

For human Tfh cell differentiation, naïve CD4^+^ T cells were isolated from PBMCs by human naïve CD4^+^ T Cell Isolation Kit (Miltenyi Biotec), and then, cells were stimulated with plate-bound anti-CD3 (5 μg/mL) and anti-CD28 (2 μg/mL) plus TGF-β (5 ng/mL) (R&D Systems), IL-6 (20 ng/mL) (PeproTech), IL-12 (10 ng/mL) (PeproTech), and IL-21 (20 ng/mL) (R&D Systems) for 3 to 5 days to induce Tfh cells.

### Treating CD4^+^ T cells with 5-azacytidine

After being stimulated for 24 h with plate-bound anti-CD3 and soluble anti-CD28 antibodies, the CD4^+^ T cells were treated with 5-azacytidine (5-aza, 5 µM, Selleck, S1782) additional 48 h. Then, DNA and RNA were extracted for DNA methylation genotyping and qRT-PCR.

### Lentiviral transduction for gene overexpression

Lentiviral transduction was used for gene overexpression to clarify the function of the *BCL2L14 gene*. The lentivirus-producing cell line (HEK293T/17, ATCC, CRL-11268™) was transiently cotransfected with the transfer plasmid (*BCL2L14*; GeneCopoeia) and packaging plasmid (GeneCopoeia, Cat. No. LT003-01) for 48 h to generate lentiviral particles. After centrifugal removal of a cellular constituent, the viral titer in the viral supernatant was increased to 1 × 10^8^ TU/ml by virus concentration (lenti-Pac^TM^Lentivirus Concentration Solution). Total CD4^+^ T or naïve CD4^+^ T cells were transfected at an MOI of 100 with IL-2 (20 ng/ml, Protein tech, 200–02-50) and polybrene (8 μg/ml, Merck, TR-1003-G). The transduction process lasted 24 h. Then, CD4^+^ T cells or naïve CD4^+^ T cells were cultured in fresh medium for 3 ~ 4 days for follow-up experiments.

### Cell coculture

The Human Renal Tubular epithelial cell line HK2 cells (ATCC, CRL-2190™) were cultured in complete DMEM/F12 Medium (Takara) containing 10% fetal bovine serum (HyClone). CD4^+^ T cells were added to HK2 cells when the fusion degree was 70%, where they could contact each other directly. The HK2 cells were collected after 24 h of coculture.

### qRT-PCR

The qRT-PCR primers used were designed using Primer 5.0 and blasted for specificity in NCBI. qRT-PCR was performed using 2 × SYBR Green PCR Master Mix (Arraystar) on an Applied Biosystems ViiA 7 Real-Time PCR System (Thermo Fisher).

### Statistical analysis

GraphPad Prism 7.0 (GraphPad Software, La Jolla, CA, USA) and SPSS version 26.0 (IBM Corp., Armonk, NY, USA) were used to analyze the data. Continuous data were presented as the means ± SD, which were compared using Student’s t test or analysis of variance for normally distributed data and the Mann–Whitney U test or Kruskal–Wallis test for skewed data. Categorical data were presented as absolute values and percentages, which were compared using Fisher’s exact chi-square test. Multivariate logistic regression analyses were performed to establish the diagnostic models and correction. The receiver operating characteristics (ROC) curve was used for the diagnostic evaluation and correction. *p* < 0.05 was considered statistically significant.

## Results

### Differential DNA methylation profiling between SLE patients and healthy controls

We extracted DNA from the CD4^+^ T cells of three groups, the LN group (mean age 32.75 ± 9.25 years), the SLE-NKI group (mean age 32.30 ± 8.41 years), and the HC group (32.25 ± 9.11 years), which included 8 cases in each group. (Patient characteristics are shown in Additional file 1: S-Table 2 and S-Table 3.)

**Fig. 1:**
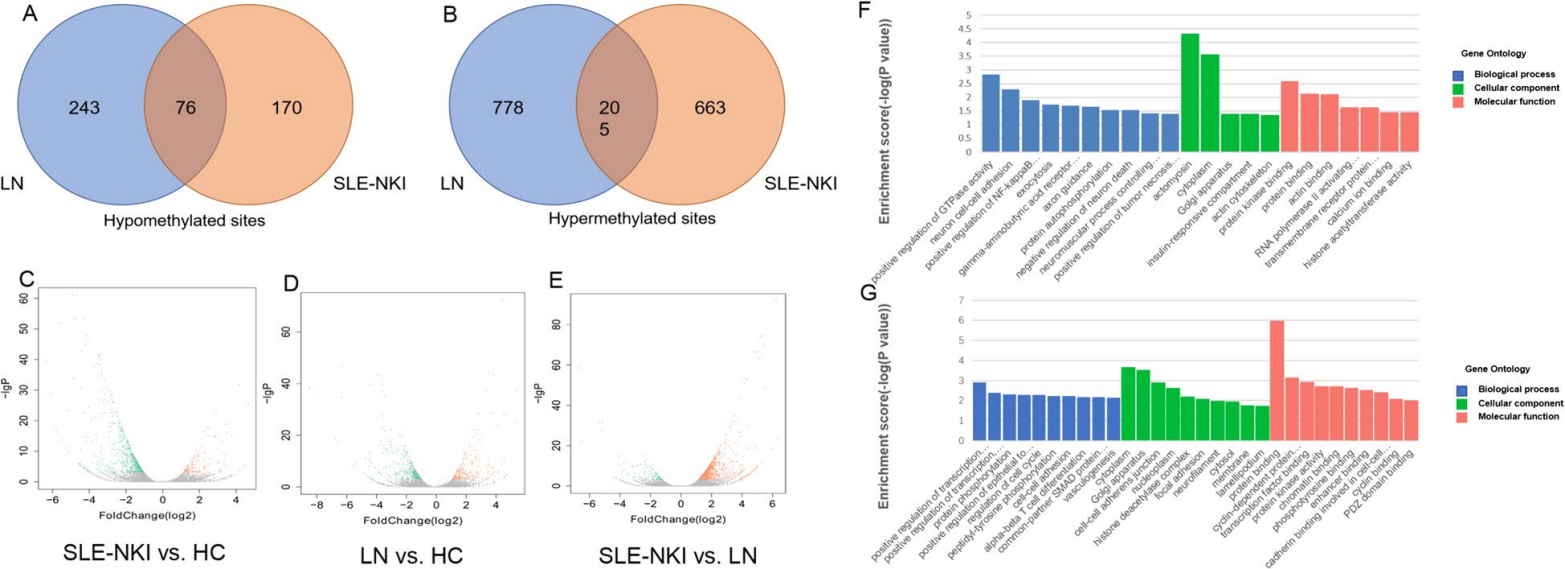
Overview of the DMPs for three groups. **A, B** Venn Diagram showing the number of hypomethylated (**A**) and hypermethylated (**B**) common and specific DMPs in LN group and SLE-NKI group compared with healthy controls. **C** The volcano plot for differential expression genes between SLE-NKI and HC; **D** The volcano plot for differential expression genes between LN and HC; **E** The volcano plot for differential expression genes between SLE-NKI and LN. **F** GO analysis for hypomethylated sites unique to LN. **G** GO analysis for hypermethylated sites unique to LN

**Fig. 2:**
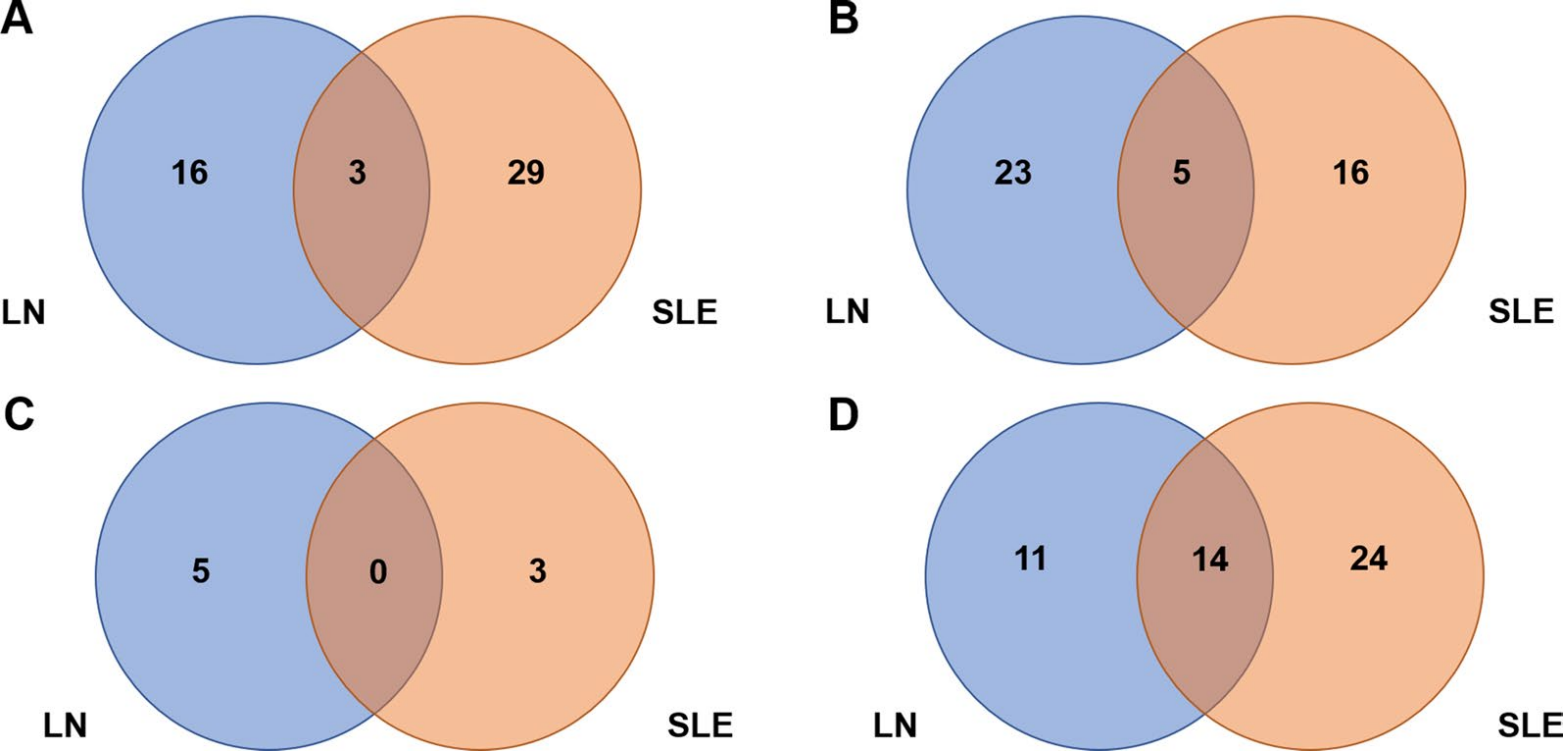
Venn diagram showing the number of differential methylated and expression genes that are common and specific in the LN group and SLE-NKI group compared with HC. **A** The hypermethylated and up-regulation genes that are common and specific in the LN group and SLE-NKI group compared with HC. **B** The hypermethylated and down-regulation genes that are common and specific in the LN group and SLE-NKI group compared with HC. **C** The hypomethylated and down-regulation genes that are common and specific in the LN group and SLE-NKI group compared with HC. **D** The hypomethylated and down-regulation genes that are common and specific in the LN group and SLE-NKI group compared with HC

**Fig. 3:**
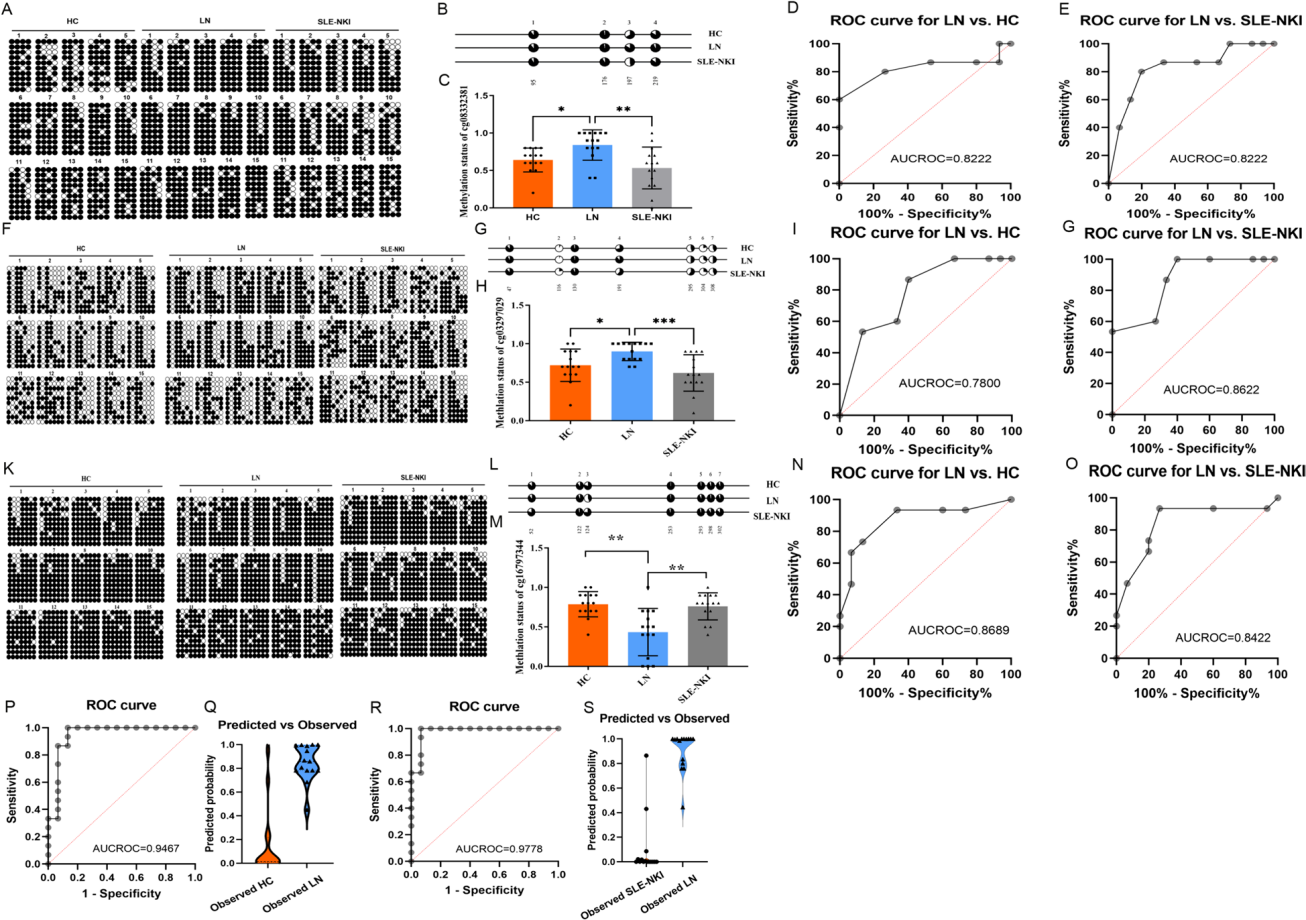
The DNA methylation condition of cg08332381, cg03297029, and cg16797344 among LN, SLE-NKI, and HC group. **A, B** Bisulfite sequencing was used to detect the methylation level of sites around cg08332381(the third point,197). **C** Mean methylation level of cg08332381 among three groups. **D, E** Receiver operator characteristic (ROC) curve showing the sensitivity and specificity of the methylation level on cg08332381for the LN group compared with HC (*p* = 0.0026) and SLE-NKI (*p* = 0.0026) group. **F, G** Bisulfite sequencing was used to detect the methylation level of sites around cg03297029 (the fourth point, 191). H: Mean methylation level of cg03297029 among three groups. **I, J** ROC curve showing the sensitivity and specificity of the methylation level on cg03297029 for the LN group compared with HC (*p* = 0.0090) and SLE-NKI (*p* = 0.0007) group. **K, L** Bisulfite sequencing was used to detect the methylation level of sites around cg16797344 (the fourth point, 191). **M** Mean methylation level of cg16797344 among three groups. **N, O** ROC curve showing the sensitivity and specificity of the methylation level on cg16797344 for the LN group compared with HC (*p* = 0.0006) and SLE-NKI (*p* = 0.0014) group. **P, Q** ROC curve and Violin Plot showing the diagnostic efficiency of the model for LN versus HC; **R, S** ROC curve and Violin Plot showing the diagnostic efficiency of the model for LN versus SLE-NKI. *p < 0.05; **p < 0.01, ***p < 0.001; *n* = 15

To investigate DNA methylation patterns in the three groups, we performed comparative analyses of methylation level data from the 3 groups. Compared with the HC group, 246 hypomethylated sites and 868 hypermethylated sites were identified in the SLE-NKI group (Additional file 2: S-Fig. 1), and 319 hypomethylated sites and 983 hypermethylated sites were identified in the LN group (Additional file 3: S-Fig. 2). Compared with SLE-NKI, 211 hypomethylated sites and 611 hypermethylated sites were identified in the LN group (Additional file 4: S-Fig. 3). In addition, 76 common hypomethylated sites (44 genes) and 205 common hypermethylated sites (113 genes) were observed in the SLE-NKI and LN groups relative to the HC group. We also identified 243 hypomethylated sites (139 genes) and 778 hypermethylated sites (455 genes) only in the LN group (Fig. 1A, B and Additional file 1: S-Table 4). The genes that are well known as biomarkers for SLE patients, such as *IFI44*, *IFI44L*, and *MX1*, and that might be differential methylated only in LN patients, such as *FGR*, *C17orf75*, and *HDAC4*, were all validated in our samples [9–11].

We analyzed the canonical pathways of differentially methylated genes only in the LN group through GO and KEGG pathway analyses. For the GO analysis of hypomethylated sites only in the LN group, ‘positive regulation of GTPase activity’ in biological process, ‘actomyosin’ in cellular components, and ‘protein kinase binding’ in molecular functions earned the highest enrichment scores. For the GO analysis of hypermethylated sites only in the LN group, ‘positive regulation of transcription from RNA polymerase II promoter’ in biological process, ‘cytoplasm’ in cellular components, and ‘protein binding’ in molecular functions earned the highest enrichment scores (Fig. 1F, G). For the KEGG analysis, the most enriched 11 pathways are shown in Table 2, which are mainly enriched in T cell receptor signaling pathways and pathways in cancer.

**Table 2:** KEGG analysis for DMPs only in the LN group

DNA methylation condition	Items	Genes	Enrichment score	*p* value
Decreased DNA methylation	Endocytosis	*RAB4A, GRK5, IQSEC, ARPC, AMPH, SMAP, IGF1R*	1.91	0.011986
	Pathways in cancer	*CSF1R, FGF7, COL4A1, LAMA4, STAT3, CCDC6, TRAF, IGF1R*	1.45	0.035783
	Transcriptional misregulation in cancer	*CSF1R, CCNT, MLLT3, SUPT3H, IGF1R*	1.36	0.043866
Increased DNA methylation	Chronic myeloid leukemia	*BCR, MAPK, CDK6, GRB, CBL, TGFBR, RUNX1*	0.32	0.003808
	T cell receptor signaling pathway	*MAPK, PTPRC, GRAP, NFATC3, NFATC1, GRB, CD47, CARD11*	0.32	0.004815
	Signaling pathways regulating pluripotency of stem cells	*FZD1, SMAD, MAPK, DLX5, KAT6A, GRB, AXIN, IL6ST, JARID*	0.05	0.008887
	Endocytosis	*SMAD, CAPZB, GRK5, WWP1, VPS37B, CBL, LDLRAP1, CHMP7, CYTH1, RAB11FIP4, RAB7A, TGFBR*	1.91	0.01005
	Pathways in cancer	*FZD1, SMAD, CREBBP, MAPK, EPAS1, LEF1, GNAI3, AXIN, CBL, ETS1, TGFBR, RUNX1, BCR, CDK6, COL4A, GRB*	1.78	0.0171
	Oxytocin signaling pathway	*GNAO1, MAPK, CAMK4, GNAI3, NFATC3, CACNAD, NFATC1, MAPK5*	1.46	0.037561
	Glycosphingolipid biosynthesis—ganglion series	*GLB1, HEXB, ST8SIA5*	1.42	0.0383
	Morphine addiction	*GNAO1, GRK5, GNAI3, PDE7B, OPRM1, PDE7A*	1.38	0.041557

### Differential gene expression profiling and the integration of DNA methylation data with mRNA transcript expression data

DNA methylation is generally associated with gene expression regulation [23, 24]. To integrate our methylation data with gene expression data, we screened high-throughput gene expression profiles in CD4^+^ T cells from the same samples for DNA methylation genotyping. Compared with the HC group, 932 down-regulation genes and 2305 up-regulated genes were identified in the SLE-NKI group; 1049 down-regulated genes and 1992 up-regulated genes were identified in the LN group (Fig. 1C, D). Compared with the LN group, 761 down-regulated genes and 2539 up-regulated genes were identified in the SLE-NKI group (Fig. 1E).

To determine the potential effects of DNA methylation on the abnormal gene expression in LN and SLE-NKI, we investigated the gene expression level of the DMPs we detected in LN and SLE-NKI groups. In the LN group, we got 19 hypermethylated and up-regulated genes, 26 hypermethylated and down-regulated genes, 25 hypomethylated and up-regulated genes, and 5 hypomethylated and down-regulated genes. In the SLE-NKI group, we got 32 hypermethylated and up-regulated genes, 21 hypermethylated and down-regulated genes, 38 hypomethylated and up-regulated genes, and 3 hypomethylated and down-regulated genes (Additional file 1: S-Table 5 and S-Table 6).

Venn diagram shows the number of common and specific differentially methylated expression sites and genes in the LN group and SLE-NKI group compared with the HC group. For the differential methylated and expression transcripts might only in the LN group, 16 hypermethylated and up-regulation transcripts were found, 23 hypermethylated and down-regulation transcripts were found, 5 hypomethylated and up-regulation transcripts were found, and 11 hypomethylated and down-regulation transcripts were found (Fig. 2). GO analysis of the differential methylated and expression genes only in the LN group showed that these genes were enriched in the apoptotic process, and negative regulation of cell proliferation.

### Validation of the DMPs only in the LN group with an external group

We got 3 DMPs (detailed information supplied in Additional file 1: S-Table 1) that were significantly methylated among the three groups (Fig. 3, Table 3). The correction analysis with age, SLEDAI index, and medications by multivariate logistic regression showed that the three DMPs showed difference between groups independent with these factors (Additional file 1: S-Table 7). The DNA methylation levels of cg08332381 (Fig. 3A–C) and cg03297029 (Fig. 3F–H) in the LN group were increased compared to the HC group and the SLE-NKI group. The area under the receiver operating characteristic curves (AUCROC) of cg08332381 methylation levels for LN versus HC reached 0.8222 (*p* = 0.0026), and for LN versus SLE-NKI, it reached 0.8222 (*p* = 0.0026) (Fig. 3D, E). The AUCROC of cg03297029 methylation levels for LN versus HC reached 0.7800 (*p* = 0.0090), and for LN versus SLE-NKI, it reached 0.8622 (*p* = 0007) (Fig. 3I, G). The DNA methylation level of cg16797344 in the LN group was lower than that compared in the HC group and SLE-NKI group (Fig. 3K–M); the AUCROC of which for LN versus HC reached 0.8689 (*p* = 0.0006), and that for LN versus SLE-NKI reached 0.8422 (*p* = 0.0014) (Fig. 3N, O). Three loci combined with multivariate logistic regression to build diagnostic models for better diagnostic efficiency and we obtained the following equations:

**Table 3:** Performance of three DMPs for distinguishing effects between groups

DMPs	Groups	Sensitivity	Specificity	AUCROC	*p* value
cg08332381	LN versus HC	0.8000	0.7333	0.8222	0.0026
	LN versus SLE-NKI	0.8000	0.8	0.8222	0.0026
cg03297029	LN versus HC	0.8667	0.6000	0.7800	0.0090
	LN versus SLE-NKI	0.8667	0.6667	0.8622	0.0007
cg16797344	LN versus HC	0.9333	0.6667	0.8689	0.0006
	LN versus SLE-NKI	0.9333	0.7333	0.8422	0.0014
Combined	LN versus HC	0.8667	0.9333	0.9467	< 0.0001
	LN versus SLE-NKI	0.9333	0.9333	0.9778	< 0.0001

P_LN_ _(LN_ versus _HC)_ = 1/[1 + exp (5.253–14.64×(cg08332381)-0.6697×(cg03297029) + 10.26×(cg16797344))/]

P_LN_ _(LN_ versus _SLE-NKI)_ = 1/[1 + exp (12.32 – 6.709×(cg08332381) − 25.33×(cg03297029)+ 18.33×(cg16797344))/]

The AUCROC of the models is all around 0.9(*P*_LN_ versus _HC_ < 0.0001; *P*_LN_ versus _SLE-NKI_ < 0.0001) (Fig. 3P–S, Table 3).

### Decreased DNA methylation increases the mRNA expression

**Fig. 4:**
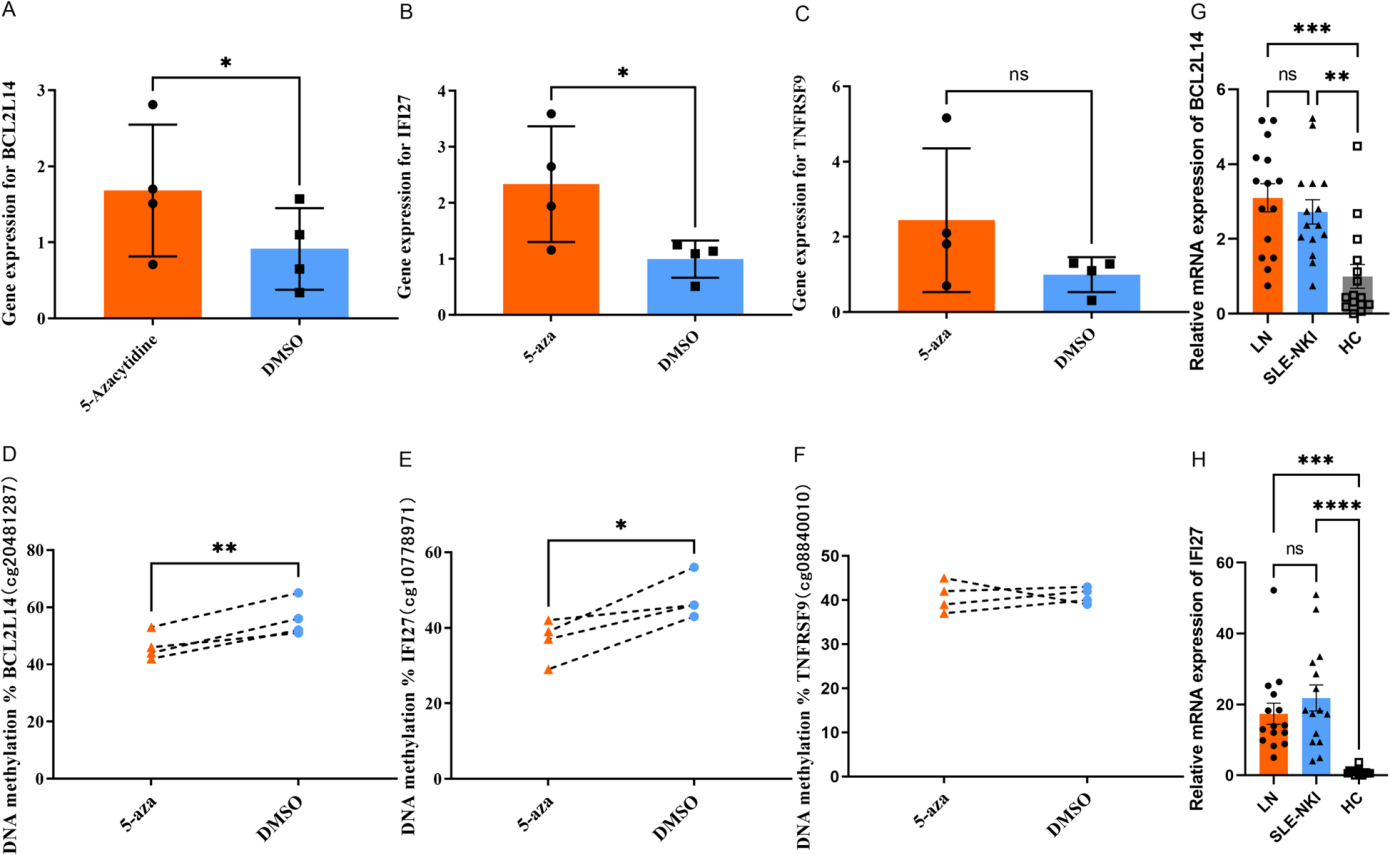
The expression of gene IFI27 and BCL2L14 might be regulated by DNA methylation. **A–C** The DNA methylation level of IFI27, BCL2L14, and TNFRSF9 in CD4^+^T cell after 5-aza treatment (*n* = 4). **D–F** The expression level of gene IFI27, BCL2L14 andTNFRSF9 in CD4^+^ T cell after 5-aza treatment (*n* = 4); **G, H** RT-qPCR for validation of selective genes in an additional 15 samples for each group. *p < 0.05, **p < 0.01, ***p < 0.001, ****p < 0.0001

Nucleoside analogue inhibitors of DNA methyltransferases, such as 5-aza-C^3^ and 5-aza-dC, have been widely used in attempts to reverse abnormal DNA methylation changes in cells [25, 26]. We randomly selected three differentially methylated and expressed genes, the DMPs of which were located in the 5’UTR, that were only in the LN group to verify whether gene expression was influenced by DNA methylation level in CD4^+^ T cells isolated from healthy donors (*n* = 4). The primers used and detailed information on these genes are listed in Additional file 1: S-Table 8. The results showed that the expression and methylation levels of *IFI27*, and *BCL2L14* all significantly changed after treatment with 5-aza, consistent with our profiling results, the DNA methylation level decreased, and the expression level increased. However, for the *TNFRS9*, the expression increased, but the DNA methylation level did not change much (Fig. 4).

We then performed quantitative RT-qPCR to investigate the expression status of *IFI27* and *BCL2L14* in the external validation groups [15 for each group]. The results confirmed that the expression levels of *IFI27* and *BCL2L14* were elevated in LN and SLE-NKI patients compared with HCs, which was consistent with the RNA-seq data (Fig. 4G, H).

### *BCL2L14* contributes to pathogenic T cell function and is associated with renal fibrosis

**Fig. 5:**
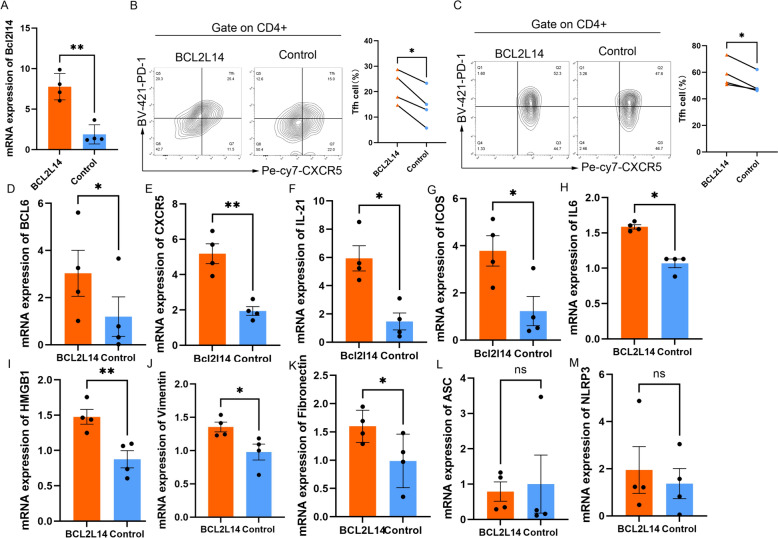
BCL2L14 overexpressed in CD4^+^T cells and cocultured with HK2 cells. **A** The expression level of in CD4^+^T cells with BCL2L14 overexpression; **B** FACS analysis of differentiation ratio of Tfh cells in CD4^+^T cells with BCL2L14 overexpression (*n* = 4, *p* = 0.0087). **C** FACS analysis of induced Tfh cells in cells transfected with BCL2L14 (*n* = 4, *p* = 0.0230). **D–G** the expression level of BCL6, CXCR5, IL-21, and ICOS level of CD4^+^T cell with BCL2L14 overexpressed; **H–M** The expression level of IL-6, HMGB1, Vimentin, Fibronectin, ASC and NLRP3 in HK2 that cocultured with CD4^+^ T cells with BCL2L14 overexpressed. *p < 0.05, **p < 0.01, *n* = 4

With the help of lentiviral transduction, the expression of *BCL2L14* was increased in healthy CD4^+^ T cells (Fig. 5A). FACS showed that the overexpression of *BCL2L14* significantly increased the differentiation ratio of Tfh cells (*p* = 0.0334, *n* = 4) but did not affect other subsets of CD4^+^ T cells (Fig. 5B, Additional file 5: Fig. 4). To confirm the role of *BCL2L14* in Tfh cell differentiation, we overexpressed *BCL2L14* in healthy naïve CD4^+^ T cells and stimulated them to differentiate into Tfh cells in vitro, the changes were same (*n* = 4, *p* = 0.0230) (Fig. 5C). Simultaneously, the mRNA levels of *BCL6*, *CXCR6*, *IL-21,* and *ICOS* were significantly increased (Fig. 5D–G).

Then, we cocultured the CD4^+^ T cells with HK2 cells for 24 h. We found that overexpression of *BCL2L14* in CD4^+^ T cells could induce fibrosis in HK2 cells. The expression of the inflammatory markers *IL-6* and *HMGB1* was significantly increased (Fig. 5H, I). The expression levels of *vimentin* and *fibronectin* were significantly induced (Fig. 5J, K). Similarly, meanwhile, the apoptosis markers, *ASC* and *NLRP3*, did not significantly change (Fig. 5L, M).

## Discussion

Our study profiled the DNA methylation in CD4^+^ T cells from the SLE-NKI, LN, and HC groups. With the help of 850 k microarray analysis, RNA-seq, and integrated analysis of DNA methylation and mRNA expression data, we established a profile of DNA methylation and mRNA expression in SLE patients, LN especially. By comparing these groups, we obtained differential DMPs only in the LN group, even genes that were differentially methylated and differentially expressed at the same time. With the help of BS-PCR, we identified several DMPs that could be potential LN biomarkers. We also found that the DNA methylation level might be an important factor that can regulate the expression of the *IFI27* and *BCL2L14* genes. Overexpression of *BCL2L14* is associated with abnormal Tfh cell function and renal fibrosis.

Epigenetic modifications are heritable changes in genome that occur without underlying modifications in the sequence. Via epigenetic mechanisms, internal and external environmental risk factors could influence the pathogenesis of autoimmune disease (27). Thus, epigenetic modification is a good bridge between environmental and genetic factors [28]. DNA methylation is a classical epigenetic modification that was discovered when DNA was confirmed as genetic material [29, 30]. Some scholars believe that DNA methylation defects in lupus patients promote an overactive immune response when they are exposed to inflammatory signals such as autoantibody–autoantigen complexes or endogenous nucleic acids [31, 32]. Thus, it is important to investigate the DNA methylation in SLE and LN patients for a better understanding of the disease and to seek diagnosis, treatment, and prognosis biomarkers. This study identified 1021 DMPs (594 genes) only in LN patients. Moreover, BS-PCR was used for external validation and revealed that cg08332381 and cg03297029 were significantly hypermethylated in the LN group compared with the SLE-NKI and HC groups, while cg16797344 was significantly hypomethylated in the LN group compared with SLE-NKI and HC group. These sites showed good potential as LN biomarkers, and the AUCROC reached around 0.8. What’s more, with the help of multivariate logistic regression, we found that the three DMPs together could identify LN more accurately, AUCROC of which could reach more than 0.9.

Cg08332381 was located in the body-opensea of *CDC42BPA*, which is a member of the serine/threonine protein kinase family that correlated with actin cytoskeleton organization and cell migration [33] and was reported implicated in several cancer types, including skin cancer, ovarian carcinoma and glioblastoma [34]. Cg03297029 was located in the body-opensea of *GRK5*, which encodes a member of the guanine nucleotide-binding protein (G protein)-coupled receptor kinase subfamily of the Ser/Thr protein kinase family. *GRK* activity fold increases when T cells are exposed to PHA [35]. And *GRK5* could induce T-lymphocytes et.al infiltration [36]. Cg16797344 was located in the Body-shelf of *ST8SIA5* which is an α2,8-sialyltransferase involved in ganglioside synthesis, which is an important component of the membrane [37]. Whether these genes are related to T cell migration and tissue invasion remains to be determined.

The addition of a methyl group onto the 5' carbon position of cytosine in cytosine-phosphate guanosine (CpG) dinucleotides can potently reduce the accessibility to DNA for transcription factors and RNA polymerases, and thereby repress transcription [38]. Integrated analysis of the DNA methylation and RNA-seq data of CD4^+^ T cells from the same groups revealed 72 genes that were consistently differentially methylated and expressed only in the LN group compared with healthy controls; these genes are likely to participate in the progression of LN. To explore whether the expression of these genes is regulated by DNA methylation. We treated CD4^+^ T cells from healthy donors (*n* = 4) with 5-aza and found that the DNA methylation status and expression of *IFI27* and *BCL2L14* changed as the sequencing analysis.

After treatment with 5-aza, cg10778971, which is located in the 5’UTR-opensea region of *IFI27*, was hypomethylated, and the expression of *IFI27* increased simultaneously. Therefore, we speculated that the expression of *IFI27* might be influenced by the methylation status of cg10778971. The expression of IFI27 in CD4^+^T cells of LN and SLE-NKI patients was externally validated in large groups, which is highly expressed compared with the HC group. Several studies have reported that *IFI27* is an immune biomarker for many autoimmune diseases, such as dermatomyositis [39], Sjögren’s syndrome [40], ANCA-associated vasculitis, SLE [41, 42], the expression of which is increased significantly, and is likely involved in the immunoregulation of these diseases [43]. There is evidence that *IFI27* expression clustered with the fraction of SLE cases having African ancestry or lupus nephritis [44], which also has growth inhibitory and anti-viral functions, including the ability to sensitize cells to apoptosis that is the same as other common ISGs [44, 45].

The same situation applies to *BCL2L14*, the expression of which is likely influenced by cg20481287 located in the 5’UTR. *BCL2L14* encodes the protein BCL-G, a nontypical protein of the BCL-2 protein family, that plays distinct roles in different types of cells and disorders [46]. By examining tissue from the kidneys of patients with lupus nephritis, Ko and colleagues discovered that kidney-infiltrating B cells and T cells, which are located mainly in the tubulointerstitial space, expressed high levels of BCl-2 in comparison to activated lymphocytes from the germinal centers of healthy secondary lymphoid tissues [47]. A similar pattern of BCL-2 was found in the NZB/WF_1_ mouse model of lupus [47]. Evidence showed that BCL-G has functions in intracellular trafficking, immunomodulation, and regulation of the mucin scaffolding network, but no apoptosis [46, 48]. Our results showed that *BCL2L14* is related to the differentiation and function of Tfh cells, which might be related to the aberrant expansion of Tfh cells, which is closely related to the progression of autoimmune diseases [49]. Tfh cells could couple with B cells to form germinal centers in kidneys [50, 51], which also could directly promote renal fibrosis by IL-21 [52]. Couple this with the fact that our results also showed that overexpression of *BCL2L14* in CD4^+^ T cells could induce fibrosis and inflammation but not apoptosis in HK2 cells.

In summary, we systematically profiled the DNA methylation and expression patterns in SLE CD4^+^ T cells of LN patients. We got three potential biomarkers for LN patients and the combined models showed good efficacy of discrimination. While, these DMPs also need to be validated in peripheral blood and large samples to figure out its practicability in clinical setting. The integration analysis of DNA methylation and expression offered some genes that might participate in the progress of LN. We also found that *BCL2L1*4 has a role in regulating Tfh cells, which is likely related to mechanism of LN. The function of *BCL2L14* will be further studied in follow-up animal experiments. Our results provide a new perspective for the research for SLE and offer more ideas for other researchers.

### Supplementary Information

**Additional file 1**.

**Additional file 2: Figure 1**. DMP between SLE-NKI group and the healthy control group. A: The volcano plot for DMP; B: Top 70 genes mostly enriched by significant CpGs. Taking the gene name as the X-axis, the number of DMP as the Y-axis. C: The distribution of DMP among chromosomes. D: The distribution of DMP in the gene.

**Additional file 3: Figure 2**. DMP between LN group and healthy control group. A: The volcano plot for DMP; B: Top 70 genes mostly enriched by significant CpGs. Taking the gene name as the X-axis, the number of DMP as the Y-axis. C: The distribution of DMP among chromosomes. D: The distribution of DMP in the gene.

**Additional file 4: Figure 3**. DMP between LN group and SLE-NKI group. A: The volcano plot for DMP; B: Top 70 genes mostly enriched by significant CpGs. Taking the gene name as the X-axis, the number of DMP as the Y-axis. C: The distribution of DMP among chromosomes. D: The distribution of DMP in the gene.

**Additional file 5: Figure 5**. FACS analysis of differentiation ratio of Treg, Th1, Th2, and Th17 cells in CD4+T cells with BCL2L14 overexpression, which all have no differences. A: Treg cells; B: Th1 cells; C: Th2 cells; D:Th17 cells.

## Data Availability

The data supporting this study’s findings are available from the corresponding author upon request.
