# Supplementary material for: DNA methylation and whole-genome transcription analysis in CD4+ T cells from systemic lupus erythematosus patients with or without renal damage
Source: Clin Epigenetics. 2024 Jul 30;16:98. doi: 10.1186/s13148-024-01699-7 (PMC11290231; doi:10.1186/s13148-024-01699-7)
Supplement: Supplementary file 1 — Additional file 1. [file 13148_2024_1699_MOESM1_ESM.docx]

| **Supplementary-table 1. The primers and annealing temperature for BS-PCR** | | | | | | | | |
| --- | --- | --- | --- | --- | --- | --- | --- | --- |
| **No.** | **Cg site** | **Gene** | **Feat#cgi** | **Outer primers** | **Annealing Temperature** | **Inner primers** | **Annealing Temperature** |  |
| Cg1 | cg08332381 | CDC42BPA | Body-opensea | F1: TCGTTGAGTTTGGTTGTGGA  R1: CGCCCAACCCAATTCACATT | 55.8℃ | F1: GGTTGGTTGTTTTTTTGGAAATAT  R: ACTAAAATAAAACTCCCATATCACTACTA | 58.8℃ |  |
| Cg2 | cg03297029 | GRK5 | Body-opensea | F1: TTTGGGATGGGGGATGTGTT  R1: AACAACAACAACAATTCCTTCCT | 55.8℃ | F1: GGGTGATTTGGTTGGTTGTATAT  R1: CCTTCCTAACTCCAAAAAACAACTCT | 58.8℃ |  |
| Cg3 | cg16797344 | ST8SIA5 | Body-shelf | F1: TTGGTTTTGAAGTGGATTTGTTTTT  R1: AACGAATTTTCAACCTATTAACCAAAA | 53.7℃ | F1: TTTTAAAATATTTTGTTGAGGTTAGTGAT  R1: CAACCTATTAACCAAAATAATCTTAATCTC | 56.5℃ |  |

| **Supplementary Table 2. Basic information for Sequencing samples** | | | | | | | | |
| --- | --- | --- | --- | --- | --- | --- | --- | --- |
| **Group** | **LN** | | | **SLE-NKI** | | | **HC** | |
|  | **Age** | **SLEDAI** | | **Age** | **SLEDAI** | | **Age** | **SLEDAI** |
| 1 | 36 | 14 | | 38 | 8 | | 40 | 0 |
| 2 | 34 | 16 | | 36 | 4 | | 32 | 0 |
| 3 | 32 | 10 | | 31 | 0 | | 29 | 0 |
| 4 | 32 | 16 | | 29 | 2 | | 28 | 0 |
| 5 | 25 | 12 | | 26 | 12 | | 25 | 0 |
| 6 | 28 | | 12 | 28 | | 4 | 26 | 0 |
| 7 | 51 | | 12 | 50 | | 2 | 51 | 0 |
| 8 | 24 | | 6 | 20 | | 4 | 24 | 0 |
| LN, lupus nephritis; SLE-NKI, systemic lupus erythematosus without kidney injury; HC, healthy control; SLEDAI, systemic lupus erythematosus disease activity index | | | | | | | | |

| **Supplementary Table 3. Clinical parameters of patients enrolled for sequencing** | | | |
| --- | --- | --- | --- |
| **Indexes** | **LN** | **SLE-NKI** | ***p-*value** |
| BMI | 23.10±5.20 | 20.40±5.12 | 0.330 |
| Hemoglobin(g/L) | 106.14±26.37 | 130.13±19.06 | 0.062 |
| blood platelet | 243.57±103.33 | 245.00±77.29 | 0.0976 |
| ALB(g/L) | 32.62±6.82 | 44.96±2.94 | 0.001 |
| SLEDAI | 12.25±3.28 | 4.50±3.82 | 0.001 |
| Proteinuria | 7（87.5%） | 0（0.0%） | P<0.001 |
| Urine leukocyte | 4（50.0%） | 0（0.0%） | 0.021 |
| Urine erythrocyte | 4（50.0%） | 0（0.0%） | 0.021 |
| Urine tube type | 0（0.0%） | 0（0.0%） | - |
| Hypertension | 2（28.6%） | 1（16.7%） | 0.612 |
| Anti-dsDNA | 0（0.0%） | 2（25.0%） | 0.155 |
| Anti-Sm | 1（14.3%） | 1（12.3%） | 0.919 |
| SS-A | 2（28.6） | 6（75.0%） | 0.072 |
| SS-B | 0（0.0%） | 1（12.3%） | 0.333 |
| Complement C3 | 0.61±0.17 | 0.91±0.27 | 0.026 |
| Complement C4 | 0.16±0.07 | 0.13±0.08 | 0.521 |
| Nucleosomes | 0（0.0%） | 1（14.3%） | 0.490 |
| Ribosomal protein | 0（0.0%） | 3（37.5%） | 0.070 |
| RO-52 | 1（16.7%） | 6（75.0%） | 0.031 |
| Erythrocyte sedimentation rate | 38.50±36.18 | 10.00±6.72 | 0.213 |
| Hydroxychloroquine sulfate | 7（87.5%） | 7（87.5%） | 1 |
| Immunosuppressant | 5(62.3%) | 3(37.5%) | 0.317 |
| Glucocorticoid | 7(87.5%) | 8(100%) | 0.302 |
| LN, lupus nephritis; SLE, systemic lupus erythematosus; BMI, body mass index; ALB, serum albumin; SLEDAI, systemic lupus erythematosus disease activity index; dsDNA, double-stranded deoxyribonucleic acid | | | |

| **Supplementary Table 4. The DMPs unique to LN group (top 30)** | | | | | | | | | | |
| --- | --- | --- | --- | --- | --- | --- | --- | --- | --- | --- |
| **List** | **DNA methylation condition** | **Gene** | **CG sites** | **Methylation Fraction(beta)** | | | **LogFC** | **Feature** | **Cgi** | ***P*-value** |
|  |  |  |  | **LN-Avarage** | **HC-Avarage** | **delta beta** |  |  |  |  |
| 1 | Up | PPP1R16A | cg10734164 | 0.340867 | 0.190264 | 0.150603 | 0.150603 | Body | island | 4.28E-05 |
| 2 |  |  | cg02738156 | 0.273393 | 0.17316 | 0.100233 | 0.100233 | IGR | opensea | 4.32E-05 |
| 3 |  | LIPC | cg21674010 | 0.73763 | 0.619278 | 0.118352 | 0.118352 | Body | opensea | 7.48E-05 |
| 4 |  | C6orf186 | cg06011067 | 0.579958 | 0.476775 | 0.103183 | 0.103183 | Body | shore | 7.55E-05 |
| 5 |  | PPP1R16A | cg00533182 | 0.4443 | 0.323569 | 0.120731 | 0.120731 | Body | island | 8.24E-05 |
| 6 |  | HMG20A | cg14584278 | 0.43069 | 0.323387 | 0.107303 | 0.107303 | 5'UTR | opensea | 8.96E-05 |
| 7 |  |  | cg14880421 | 0.271755 | 0.160146 | 0.111609 | 0.111609 | IGR | opensea | 9.03E-05 |
| 8 |  |  | cg00931181 | 0.706224 | 0.582608 | 0.123616 | 0.123616 | IGR | opensea | 9.13E-05 |
| 9 |  | KIAA1949 | cg26004235 | 0.653876 | 0.547273 | 0.106603 | 0.106603 | TSS1500 | shore | 0.000113 |
| 10 |  | LIPC | cg05337681 | 0.734412 | 0.604506 | 0.129905 | 0.129905 | Body | opensea | 0.000118 |
| 11 |  | FBXO38 | cg26228218 | 0.693581 | 0.576946 | 0.116635 | 0.116635 | 5'UTR | shelf | 0.000132 |
| 12 |  | PITPNC1 | cg15993159 | 0.834338 | 0.711815 | 0.122523 | 0.122523 | Body | opensea | 0.000143 |
| 13 |  | SPATS2L | cg14106933 | 0.733445 | 0.627579 | 0.105866 | 0.105866 | 5'UTR | opensea | 0.000146 |
| 14 |  |  | cg21556040 | 0.708218 | 0.586217 | 0.122001 | 0.122001 | IGR | opensea | 0.000149 |
| 15 |  |  | cg11173447 | 0.685506 | 0.488057 | 0.19745 | 0.19745 | IGR | opensea | 0.000168 |
| 16 |  |  | cg09748946 | 0.582927 | 0.482232 | 0.100695 | 0.100695 | IGR | island | 0.000189 |
| 17 |  | 44448 | cg20557159 | 0.718876 | 0.556262 | 0.162614 | 0.162614 | Body | shelf | 0.000205 |
| 18 |  | ANKRD44 | cg01418945 | 0.753223 | 0.639998 | 0.113224 | 0.113224 | Body | opensea | 0.00023 |
| 19 |  |  | cg02880119 | 0.662067 | 0.559514 | 0.102553 | 0.102553 | IGR | opensea | 0.000238 |
| 20 |  |  | cg25267863 | 0.421707 | 0.310107 | 0.111599 | 0.111599 | IGR | island | 0.000239 |
| 21 |  | GALNT6 | cg21253043 | 0.511407 | 0.396272 | 0.115135 | 0.115135 | 5'UTR | shore | 0.000244 |
| 22 |  |  | cg05686551 | 0.565775 | 0.422687 | 0.143089 | 0.143089 | IGR | opensea | 0.000264 |
| 23 |  | PHACTR3 | cg03325394 | 0.803633 | 0.481436 | 0.322197 | 0.322197 | Body | opensea | 0.000273 |
| 24 |  | ERCC6L | cg08304428 | 0.655248 | 0.553266 | 0.101982 | 0.101982 | TSS1500 | shore | 0.000286 |
| 25 |  |  | cg24447756 | 0.637754 | 0.528592 | 0.109163 | 0.109163 | IGR | opensea | 0.000288 |
| 26 |  | PHLDB3 | cg08985826 | 0.765331 | 0.656009 | 0.109322 | 0.109322 | TSS1500 | shore | 0.000333 |
| 27 |  | KIAA2012 | cg04645399 | 0.576035 | 0.463046 | 0.11299 | 0.11299 | Body | opensea | 0.000372 |
| 28 |  |  | cg04836949 | 0.286402 | 0.184604 | 0.101798 | 0.101798 | IGR | opensea | 0.000376 |
| 29 |  |  | cg24792317 | 0.811213 | 0.673795 | 0.137417 | 0.137417 | IGR | shelf | 0.000393 |
| 30 |  |  | cg23625422 | 0.618504 | 0.445081 | 0.173423 | 0.173423 | IGR | opensea | 0.0004 |
| 1 | Down | BISPR | cg07362849 | 0.046016 | 0.149911 | -0.103896 | -0.103896 | Body | opensea | 1.41E-05 |
| 2 |  | ORMDL3 | cg12749226 | 0.42009 | 0.560996 | -0.140907 | -0.140907 | 3'UTR | opensea | 4.76E-05 |
| 3 |  | BISPR | cg22337964 | 0.096619 | 0.236138 | -0.139518 | -0.139518 | Body | opensea | 0.000165 |
| 4 |  | IFI27 | cg10778971 | 0.731817 | 0.843483 | -0.111666 | -0.111666 | 5'UTR | opensea | 0.000243 |
| 5 |  | AKAP13 | cg18088723 | 0.204731 | 0.395327 | -0.190595 | -0.190595 | Body | opensea | 0.000261 |
| 6 |  | C17orf75 | cg04936619 | 0.215968 | 0.414057 | -0.198089 | -0.198089 | 3'UTR | opensea | 0.000323 |
| 7 |  | FKBP5 | cg14642437 | 0.810054 | 0.918619 | -0.108565 | -0.108565 | 5'UTR | shelf | 0.000383 |
| 8 |  | PARP14 | cg14750551 | 0.8267 | 0.9293 | -0.1026 | -0.1026 | Body | shore | 0.000384 |
| 9 |  |  | cg09681675 | 0.746196 | 0.857769 | -0.111573 | -0.111573 | IGR | opensea | 0.000405 |
| 10 |  | SLC35F4 | cg18444006 | 0.373132 | 0.522881 | -0.149749 | -0.149749 | Body | opensea | 0.000584 |
| 11 |  |  | cg25070075 | 0.646375 | 0.781296 | -0.134921 | -0.134921 | IGR | island | 0.00082 |
| 12 |  |  | cg17489610 | 0.519135 | 0.643256 | -0.124121 | -0.124121 | IGR | opensea | 0.000841 |
| 13 |  | CDC42BPA | cg08332381 | 0.477871 | 0.884483 | -0.406612 | -0.406612 | Body | opensea | 0.001238 |
| 14 |  | GRK5 | cg03297029 | 0.625227 | 0.921599 | -0.296372 | -0.296372 | Body | opensea | 0.001445 |
| 15 |  |  | cg05275368 | 0.266159 | 0.378205 | -0.112046 | -0.112046 | IGR | opensea | 0.001474 |
| 16 |  | MIA3 | cg21816128 | 0.205997 | 0.333168 | -0.127172 | -0.127172 | Body | opensea | 0.00166 |
| 17 |  | DLGAP2 | cg15833940 | 0.640002 | 0.757389 | -0.117387 | -0.117387 | Body | opensea | 0.001805 |
| 18 |  | TRIM22 | cg26724018 | 0.110497 | 0.217152 | -0.106655 | -0.106655 | 5'UTR | opensea | 0.001825 |
| 19 |  | BICC1 | cg15509952 | 0.709133 | 0.822041 | -0.112908 | -0.112908 | Body | opensea | 0.002015 |
| 20 |  |  | cg20757478 | 0.384697 | 0.857096 | -0.472399 | -0.472399 | IGR | opensea | 0.002174 |
| 21 |  |  | cg15535896 | 0.544196 | 0.914783 | -0.370587 | -0.370587 | IGR | shelf | 0.00227 |
| 22 |  | ESF1 | cg10749624 | 0.656384 | 0.805397 | -0.149012 | -0.149012 | Body | opensea | 0.002272 |
| 23 |  | INPP4B | cg02563636 | 0.457498 | 0.633996 | -0.176498 | -0.176498 | 5'UTR | opensea | 0.002364 |
| 24 |  | MYH10 | cg26999053 | 0.275262 | 0.598046 | -0.322784 | -0.322784 | Body | opensea | 0.002421 |
| 25 |  |  | cg13473803 | 0.277031 | 0.436746 | -0.159715 | -0.159715 | IGR | shelf | 0.002553 |
| 26 |  | TRAPPC9 | cg20511966 | 0.6739 | 0.962198 | -0.288298 | -0.288298 | Body | shore | 0.002732 |
| 27 |  | MIR5095 | cg09730359 | 0.544707 | 0.648181 | -0.103474 | -0.103474 | Body | opensea | 0.002757 |
| 28 |  |  | cg04128669 | 0.643811 | 0.775275 | -0.131464 | -0.131464 | IGR | opensea | 0.002786 |
| 29 |  | KAT2B | cg01967102 | 0.130963 | 0.238682 | -0.107718 | -0.107718 | Body | opensea | 0.002862 |
| 30 |  |  | cg02889356 | 0.640007 | 0.748931 | -0.108924 | -0.108924 | IGR | shelf | 0.003343 |

| **S-Table 5 correlation of gene expression and DNA methylation in LN group and SLE group** | | | | | | |
| --- | --- | --- | --- | --- | --- | --- |
| **list** | **Gene** | **DNA methylation status** | | | **Expression level** | |
|  |  | **DMP** | **Delta Beta** | ***P*-value** | **FC** | ***P*-value** |
| Hypermethylation+upregulation | | | | | | |
| 1 | LMNA | cg15750163 | 0.112414201 | 0.0000438 | 1.616139376 | 2.17E-02 |
| 2 | SPATS2L | cg14106933 | 0.10586615 | 0.000146116 | 3.29117329 | 2.04E-06 |
| 3 | C1orf170 | cg05505459 | 0.100976152 | 0.000333922 | 2.357366353 | 9.76E-04 |
| 4 | CACNA2D2 | cg00383793 | 0.102427711 | 0.000721537 | 1.837720713 | 1.48E-02 |
| 5 | FADS2 | cg01556593 | 0.105490934 | 0.000910268 | 1.869874464 | 3.95E-03 |
| 6 | SMIM18 | cg14933163 | 0.115434537 | 0.023473134 | 1.574440302 | 2.50E-02 |
| 7 |  | cg14642210 | 0.120054591 | 0.00168878 | 1.809685932 | 4.96E-02 |
| 8 | MFSD7 | cg19427345 | 0.161453879 | 0.002704735 | 1.540886033 | 2.77E-02 |
| 9 | SEC61G | cg00945507 | 0.114167234 | 0.003611068 | 1.530760515 | 4.02E-02 |
| 10 | CITED1 | cg05449830 | 0.107819697 | 0.004632495 | 2.815469007 | 3.57E-02 |
| 11 | KIF19 | cg05571310 | 0.20378171 | 0.005577268 | 2.167481335 | 1.02E-02 |
| 12 | PLEKHG7 | cg12143417 | 0.100469441 | 0.011266013 | 5.407057311 | 1.08E-02 |
| 13 | ANKRD33B | cg06762111 | 0.193665243 | 0.012615861 | 1.598866072 | 4.65E-02 |
| 14 | ZFP36 | cg10575067 | 0.154575266 | 0.015271957 | 2.105259634 | 6.93E-04 |
| 15 | MYOM2 | cg22020784 | 0.101352724 | 0.018706493 | 2.126670873 | 8.26E-04 |
| 16 | CFL2 | cg10519437 | 0.104657293 | 0.019493393 | 2.219787388 | 9.31E-04 |
| 17 | CDT1 | cg11678960 | 0.115045121 | 0.025442084 | 1.809685932 | 4.96E-02 |
| 18 | ANXA2 | cg27554954 | 0.103333668 | 0.026828017 | 1.59361627 | 3.46E-02 |
| 19 | NRG1 | cg06211434 | 0.236875976 | 0.028065771 | 8.545476672 | 2.41E-11 |
| 20 | ICAM5 | cg10604476 | 0.100708178 | 0.031545503 | 1.865885807 | 4.96E-02 |
| **Hypermethylation+downregulation** | | | | | | |
| 21 | ERCC6L | cg08304428 | 0.101982197 | 0.00028567 | 0.557769864 | 1.13E-02 |
| 22 | RBM20 | cg13942283 | 0.171335203 | 0.000366835 | 0.501967643 | 2.25E-02 |
| 23 |  | cg13074892 | 0.129206497 | 0.028408152 | 0.501967643 | 2.25E-02 |
| 24 | DZIP3 | cg19400179 | 0.132661264 | 0.000547536 | 0.625558341 | 2.30E-02 |
| 25 |  | cg18959279 | 0.103797559 | 0.002166954 | 0.625558341 | 2.30E-02 |
| 26 | MMRN1 | cg21092324 | 0.113436378 | 0.001049041 | 0.435126715 | 7.45E-03 |
| 27 |  | cg09923234 | 0.103183859 | 0.001052034 | 0.435126715 | 7.45E-03 |
| 28 | SYNJ2 | cg18758976 | 0.10878182 | 0.00114715 | 0.514372949 | 2.56E-03 |
| 29 | PLXDC1 | cg14952878 | 0.147016689 | 0.001508279 | 0.539707284 | 4.31E-03 |
| 30 | UMODL1 | cg06750670 | 0.11984753 | 0.018006964 | 0.455279888 | 1.13E-02 |
| 31 |  | cg19766460 | 0.16835849 | 0.004972313 | 0.455279888 | 1.13E-02 |
| 32 |  | cg09727148 | 0.148036274 | 0.002614956 | 0.455279888 | 1.13E-02 |
| 33 | EPHA1-AS1 | cg01748326 | 0.101505187 | 0.003835242 | 0.561459382 | 1.31E-02 |
| 34 |  | cg14514510 | 0.291301606 | 0.002922466 | 0.561459382 | 1.31E-02 |
| 35 | MTUS1 | cg01993952 | 0.138960097 | 0.003055158 | 0.580797892 | 1.24E-02 |
| 36 | ZNF215 | cg08639616 | 0.108825395 | 0.010273752 | 0.259103756 | 6.87E-05 |
| 37 | NDFIP2 | cg11770323 | 0.100479923 | 0.010803737 | 0.564149419 | 1.36E-02 |
| 38 | PDE7B | cg14623715 | 0.12081082 | 0.012688269 | 0.432624807 | 5.50E-03 |
| 39 | ACTN1 | cg08864710 | 0.111372408 | 0.01290225 | 0.634367658 | 3.37E-02 |
| 40 |  | cg00739471 | 0.132860162 | 0.013552698 | 0.634367658 | 3.37E-02 |
| 41 | NUCB2 | cg02075820 | 0.115491838 | 0.013902768 | 0.604421163 | 1.92E-02 |
| 42 | EDAR | cg19792189 | 0.113836992 | 0.014307308 | 0.622006197 | 3.29E-02 |
| 43 | DDR1 | cg14279856 | 0.108949189 | 0.014406085 | 0.600977866 | 1.97E-02 |
| 44 | GIMAP8 | cg01364581 | 0.117560314 | 0.014732334 | 0.609076482 | 2.15E-02 |
| 45 | CHI3L2 | cg02590572 | 0.107541487 | 0.019557712 | 0.511995992 | 1.66E-03 |
| 46 |  | cg14414943 | 0.104657486 | 0.01604361 | 0.511995992 | 1.66E-03 |
| 47 | SARDH | cg03371378 | 0.114843096 | 0.045795692 | 0.637829481 | 4.39E-02 |
| 48 |  | cg17346231 | 0.147148602 | 0.01639363 | 0.637829481 | 4.39E-02 |
| 49 |  | cg01490728 | 0.130302691 | 0.018152865 | 0.637829481 | 4.39E-02 |
| 50 | LRRN3 | cg06545367 | 0.123414748 | 0.018309053 | 0.381020444 | 1.10E-05 |
| 51 | CAMK4 | cg11189660 | 0.102510162 | 0.018595344 | 0.650355854 | 4.48E-02 |
| 52 | ABLIM1 | cg23786209 | 0.103834854 | 0.001287202 | 0.629062401 | 2.98E-02 |
| 53 |  | cg09145734 | 0.188131987 | 0.041265722 | 0.629062401 | 2.98E-02 |
| 54 |  | cg05634779 | 0.108654448 | 0.024010458 | 0.629062401 | 2.98E-02 |
| 55 |  | cg11115176 | 0.107109012 | 0.019669075 | 0.629062401 | 2.98E-02 |
| 56 | ZNF890P | cg22618042 | 0.143420402 | 0.02256976 | 0.532136759 | 4.85E-03 |
| 57 | PIP5K1B | cg02570721 | 0.132676025 | 0.029457938 | 0.663768489 | 2.88E-02 |
| 58 | AFF3 | cg23522480 | 0.111632843 | 0.030207921 | 0.493244958 | 1.22E-03 |
| 59 | WBSCR27 | cg20311868 | 0.111675391 | 0.031104582 | 0.423323848 | 2.98E-03 |
| **Hypomethylation+upregulation** | | | | | | |
| 60 | IFIT3 | cg06188083 | 0.125216103 | 0.002528032 | 3.108997407 | 2.24E-07 |
| 61 | RSAD2 | cg15839328 | 0.142282702 | 0.003109387 | 2.404549146 | 6.21E-04 |
| 62 |  | cg10771443 | 0.128605912 | 0.003816526 | 2.404549146 | 6.21E-04 |
| 63 | IFI44 | cg01079652 | 0.127404011 | 0.005106602 | 3.062845328 | 7.64E-07 |
| 64 | FCN1 | cg16816400 | 0.148720506 | 0.011578832 | 1.784602548 | 6.30E-03 |
| 65 | IL13RA1 | cg03823458 | 0.137112175 | 0.017265148 | 1.501273765 | 2.96E-02 |
| 66 | TNFRSF9 | cg08840010 | 0.101745354 | 0.022812744 | 1.968951476 | 1.53E-02 |
| 67 | MX1 | cg13155430 | 0.124756306 | 0.025219805 | 2.710103608 | 7.38E-06 |
| 68 |  | cg22862003 | 0.222039055 | 0.0000214 | 2.710103608 | 7.38E-06 |
| 69 |  | cg21549285 | 0.271253756 | 0.0000294 | 2.710103608 | 7.38E-06 |
| 70 | BHLHE40 | cg26269881 | 0.125464023 | 0.026487334 | 2.414869937 | 1.63E-04 |
| 71 | BCL2L14 | cg20481287 | 0.103114292 | 0.026610621 | 5.754727368 | 1.00E-09 |
| 72 | CHN2 | cg03040915 | 0.191713761 | 0.036893879 | 1.587440099 | 1.89E-02 |
| 73 | FAM13A | cg08232712 | 0.105062485 | 0.041009302 | 3.274822721 | 1.07E-07 |
| 74 | FADS2 | cg14127016 | 0.230040868 | 0.045772739 | 1.869874464 | 3.95E-03 |
| 75 | BTG3 | cg13419231 | 0.156744816 | 0.046955577 | 1.57394795 | 1.74E-02 |
| 76 | MYOM2 | cg01012295 | 0.120512148 | 0.049172108 | 2.126670873 | 8.26E-04 |
| 77 | IFI44L | cg17980508 | 0.150446461 | 0.000276619 | 4.425619592 | 1.47E-10 |
| 78 |  | cg22012079 | 0.274874327 | 0.0000479 | 4.425619592 | 1.47E-10 |
| 79 |  | cg03607951 | 0.202169259 | 0.0000518 | 4.425619592 | 1.47E-10 |
| 80 |  | cg05696877 | 0.263904172 | 2.18E-08 | 4.425619592 | 1.47E-10 |
| 81 |  | cg13452062 | 0.429185659 | 2.58E-08 | 4.425619592 | 1.47E-10 |
| 82 | PARP9 | cg07815522 | 0.24740763 | 0.00000136 | 1.676537815 | 1.91E-02 |
| 83 |  | cg00959259 | 0.25999356 | 0.0000014 | 1.676537815 | 1.91E-02 |
| 84 |  | cg22930808 | 0.272570528 | 0.00000209 | 1.676537815 | 1.91E-02 |
| 85 |  | cg08122652 | 0.227490474 | 0.00000281 | 1.676537815 | 1.91E-02 |
| 86 | LY6E | cg12110437 | 0.130664466 | 0.000816915 | 2.302069733 | 2.12E-04 |
| 87 |  | cg14392283 | 0.255130677 | 0.0000059 | 2.302069733 | 2.12E-04 |
| 88 | CMPK2 | cg01028142 | 0.191655328 | 0.0000109 | 3.302762396 | 3.04E-07 |
| 89 | LGALS9 | cg11016993 | 0.157009314 | 0.0000237 | 1.967012507 | 2.64E-03 |
| 90 | PLSCR1 | cg18686270 | 0.168806306 | 0.001227512 | 1.692812431 | 3.54E-02 |
| 91 |  | cg06981309 | 0.165308058 | 0.0000356 | 1.692812431 | 3.54E-02 |
| 92 | USP18 | cg14293575 | 0.251686817 | 0.0000653 | 3.162710814 | 3.34E-06 |
| 93 | IFIT1 | cg05552874 | 0.160542157 | 0.0000667 | 2.521574904 | 1.07E-04 |
| 94 | EPSTI1 | cg12439472 | 0.222287206 | 0.000186175 | 2.226295341 | 3.53E-04 |
| 95 | IFI27 | cg10778971 | 0.111665959 | 0.000242874 | 10.7679039 | 2.13E-19 |
| 96 | EIF2AK2 | cg14126601 | 0.143705859 | 0.00066488 | 1.763838905 | 9.26E-03 |
| **Hypomethylation+downregulation** | | | | | | |
| 97 | IMPG2 | cg05247193 | -0.10838282 | 0.014193696 | 6.30E-01 | 2.59E-02 |
| 98 | RNF144B | cg09234453 | -0.28304405 | 0.011025357 | 6.29E-01 | 3.91E-02 |
| 99 |  | cg14838562 | -0.17717468 | 0.027243662 | 6.29E-01 | 3.91E-02 |
| 100 | PTPDC1 | cg08398749 | -0.11282715 | 0.018835063 | 5.31E-01 | 5.05E-03 |
| 101 | SYNJ2 | cg00602930 | -0.27421881 | 0.032965945 | 5.14E-01 | 2.56E-03 |
| 102 | ZBTB16 | cg25345365 | -0.3184531 | 1.83E-09 | 5.37E-01 | 7.34E-03 |

| **S-Table 6 correlation of gene expression and DNA methylation in SLE-NKI group** | | | | | | |
| --- | --- | --- | --- | --- | --- | --- |
| **list** | **Gene** | **DNA methylation status** | | | **Expression level** | |
|  |  | **DMP** | **delta Beta** | **P-value** | **FC** | **P-value** |
| **Hypermethylation+upregulation** | | | | | | |
| 1 | HELZ2 | cg08227465 | 0.100031 | 1.45E-05 | 3.221437 | 4.11E-07 |
| 2 |  | cg00480970 | 0.107885 | 6.17E-05 | 3.221437 | 4.11E-07 |
| 3 | OAS1 | cg04708790 | 0.110158 | 3.85E-05 | 4.757014 | 6.62E-11 |
| 4 | NOS2 | cg23346716 | 0.103786 | 7.09E-05 | 2.912438 | 1.56E-03 |
| 5 | CHST15 | cg01825287 | 0.143565 | 0.012331 | 1.723643 | 8.84E-03 |
| 6 |  | cg24069444 | 0.104762 | 0.006551 | 1.723643 | 8.84E-03 |
| 7 |  | cg12735100 | 0.156978 | 0.000511 | 1.723643 | 8.84E-03 |
| 8 |  | cg23359561 | 0.179681 | 0.000124 | 1.723643 | 8.84E-03 |
| 9 | ANKRD33B | cg06762111 | 0.168768 | 0.014161 | 1.704699 | 2.81E-02 |
| 10 | NRG1 | cg06211434 | 0.259922 | 0.017129 | 4.553488 | 2.04E-09 |
| 11 | PRR16 | cg08796299 | 0.119274 | 0.017144 | 6.264753 | 5.63E-04 |
| 12 | CD86 | cg01436254 | 0.106736 | 0.000202 | 2.575643 | 1.59E-05 |
| 13 | CYBB | cg21065784 | 0.100581 | 0.000517 | 2.293885 | 2.09E-04 |
| 14 | WNT5B | cg05811936 | 0.111867 | 0.001961 | 4.693819 | 5.61E-07 |
| 15 | RUFY4 | cg01712671 | 0.102677 | 0.004087 | 2.861165 | 5.48E-06 |
| 16 |  | cg19902229 | 0.10139 | 0.002572 | 2.861165 | 5.48E-06 |
| 17 | LINC00487 | cg03530427 | 0.29902 | 0.003098 | 8.716944 | 1.54E-15 |
| 18 | MFSD7 | cg19427345 | 0.161543 | 0.003745 | 2.028714 | 1.26E-03 |
| 19 | DHX58 | cg00647820 | 0.139981 | 0.004377 | 1.892313 | 5.87E-03 |
| 20 | TCERG1L | cg08161061 | 0.104074 | 0.00561 | 3.839056 | 1.53E-02 |
| 21 | SLC1A7 | cg07793724 | 0.130932 | 0.00689 | 1.695611 | 4.20E-02 |
| 22 | DLGAP1 | cg25439807 | 0.136999 | 0.009495 | 5.195242 | 3.84E-10 |
| 23 | KCNJ15 | cg19788186 | 0.121279 | 0.010214 | 2.315548 | 7.81E-04 |
| 24 | CDK14 | cg26441104 | 0.140268 | 0.010375 | 3.59351 | 8.59E-07 |
| 25 | AGRN | cg13100600 | 0.10718 | 0.010748 | 3.048871 | 3.51E-06 |
| 26 | WNT6 | cg13903421 | 0.108514 | 0.010748 | 3.004616 | 1.30E-03 |
| 27 | IQSEC3 | cg24319171 | 0.131627 | 0.013315 | 1.728516 | 3.47E-02 |
| 28 | TMEM132D | cg12977384 | 0.110333 | 0.02023 | 16.11941 | 5.70E-04 |
| 29 | APLP2 | cg25354657 | 0.105398 | 0.021571 | 1.57111 | 4.26E-02 |
| 30 | TTBK1 | cg01749249 | 0.102696 | 0.022892 | 6.271148 | 1.86E-05 |
| 31 | FAM13A | cg24524716 | 0.130433 | 0.024702 | 2.565256 | 2.67E-05 |
| 32 | LILRA3 | cg21898358 | 0.269267 | 0.026548 | 4.592693 | 1.33E-10 |
| 33 |  | cg16602091 | 0.27779 | 0.02712 | 4.592693 | 1.33E-10 |
| 34 | SLC30A8 | cg04355432 | 0.114696 | 0.029149 | 10.0244 | 1.08E-02 |
| 35 | TNS1 | cg24090628 | 0.224177 | 0.036436 | 1.603792 | 4.12E-02 |
| 36 | ZEB2 | cg17757894 | 0.113662 | 0.038567 | 1.591594 | 3.81E-02 |
| 37 | TNFRSF21 | cg13523038 | 0.105054 | 0.040156 | 1.760947 | 1.68E-02 |
| 38 | UBE2E2 | cg02456060 | 0.159902 | 0.044906 | 1.734077 | 9.61E-03 |
| **Hypermethylation+downregulation** | | | | | | |
| 39 | DZIP3 | cg19400179 | 0.14677 | 8.59E-05 | 0.589645 | 1.69E-02 |
| 40 | ARHGEF4 | cg09774741 | 0.102091 | 0.000443 | 0.4403 | 8.26E-04 |
| 41 |  | cg23415434 | 0.104341 | 0.000176 | 0.4403 | 8.26E-04 |
| 42 | LMO7 | cg13134634 | 0.156365 | 0.013685 | 0.629815 | 4.32E-02 |
| 43 | CSGALNACT1 | cg03651021 | 0.110636 | 0.004297 | 0.634561 | 4.71E-02 |
| 44 |  | cg21563950 | 0.100793 | 0.013746 | 0.634561 | 4.71E-02 |
| 45 | MTUS1 | cg01993952 | 0.137229 | 0.016484 | 0.524826 | 5.03E-03 |
| 46 | LINC00299 | cg20781401 | 0.149733 | 0.000753 | 0.529746 | 7.98E-03 |
| 47 | ATHL1 | cg15747436 | 0.11518 | 0.005736 | 0.638263 | 4.72E-02 |
| 48 |  | cg04412883 | 0.129625 | 0.002234 | 0.638263 | 4.72E-02 |
| 49 | SYNJ2 | cg18758976 | 0.12162 | 0.002639 | 0.592698 | 2.41E-02 |
| 50 | RBM20 | cg13942283 | 0.120217 | 0.002745 | 0.458084 | 8.32E-03 |
| 51 | PLCB4 | cg00709948 | 0.314201 | 0.005678 | 0.489552 | 2.34E-02 |
| 52 | ZNF890P | cg22618042 | 0.163309 | 0.007186 | 0.484233 | 1.94E-03 |
| 53 | COL5A3 | cg17713488 | 0.147692 | 0.008527 | 0.62511 | 4.05E-02 |
| 54 | TPD52 | cg14587889 | 0.142052 | 0.011326 | 0.62976 | 4.37E-02 |
| 55 | ADARB2 | cg25274844 | 0.110523 | 0.020096 | 0.364125 | 4.70E-05 |
| 56 |  | cg19416590 | 0.108308 | 0.012987 | 0.364125 | 4.70E-05 |
| 57 | FAM189A2 | cg12219752 | 0.129764 | 0.013484 | 0.281128 | 3.50E-02 |
| 58 | GPR55 | cg16382047 | 0.123059 | 0.01823 | 0.581059 | 1.40E-02 |
| 59 | ARHGEF10 | cg14831281 | 0.110708 | 0.026902 | 0.492953 | 2.99E-03 |
| 60 | SYTL2 | cg21518997 | 0.126743 | 0.031922 | 0.603691 | 2.67E-02 |
| 61 | ADAM21 | cg02092632 | 0.259151 | 0.041268 | 0.415693 | 4.21E-02 |
| 62 | IL5RA | cg23032421 | 0.101538 | 0.041703 | 0.38746 | 1.02E-03 |
| 63 | CUBN | cg24571822 | 0.100254 | 0.044607 | 0.637734 | 4.57E-02 |
| **Hypomethylation+upregulation** | | | | | | |
| 64 | IFI44L | cg17980508 | -0.22762 | 4.91E-06 | 10.46818 | 1.62E-21 |
| 65 |  | cg03607951 | -0.27941 | 1.59E-06 | 10.46818 | 1.62E-21 |
| 66 |  | cg22012079 | -0.36156 | 1.06E-07 | 10.46818 | 1.62E-21 |
| 67 |  | cg13452062 | -0.47603 | 5.09E-10 | 10.46818 | 1.62E-21 |
| 68 |  | cg05696877 | -0.2832 | 5.37E-10 | 10.46818 | 1.62E-21 |
| 69 | MX1 | cg13155430 | -0.24879 | 0.000046 | 5.776971 | 1.25E-13 |
| 70 |  | cg21549285 | -0.37773 | 4.84E-07 | 5.776971 | 1.25E-13 |
| 71 |  | cg22862003 | -0.27293 | 6.27E-08 | 5.776971 | 1.25E-13 |
| 72 |  | cg26312951 | -0.12512 | 1.4E-06 | 5.776971 | 1.25E-13 |
| 73 | DDX60 | cg06650861 | -0.17246 | 0.003007 | 2.999686 | 2.16E-06 |
| 74 |  | cg05883128 | -0.12408 | 0.000115 | 2.999686 | 2.16E-06 |
| 75 |  | cg24678928 | -0.28907 | 4.77E-07 | 2.999686 | 2.16E-06 |
| 76 | PARP9 | cg07815522 | -0.27833 | 1.09E-05 | 2.721692 | 1.33E-05 |
| 77 |  | cg08122652 | -0.26206 | 5.86E-07 | 2.721692 | 1.33E-05 |
| 78 |  | cg00959259 | -0.31221 | 8.24E-07 | 2.721692 | 1.33E-05 |
| 79 |  | cg22930808 | -0.33554 | 1.46E-06 | 2.721692 | 1.33E-05 |
| 80 | ODF3B | cg11224765 | -0.10705 | 0.001677 | 3.657984 | 8.94E-09 |
| 81 |  | cg19188021 | -0.12122 | 4.82E-06 | 3.657984 | 8.94E-09 |
| 82 | LGALS9 | cg11016993 | -0.21437 | 5.19E-06 | 2.906827 | 4.06E-06 |
| 83 | PLSCR1 | cg18686270 | -0.18224 | 0.000266 | 3.231411 | 1.44E-06 |
| 84 |  | cg06981309 | -0.2286 | 1.28E-05 | 3.231411 | 1.44E-06 |
| 85 | CMPK2 | cg14595557 | -0.13125 | 0.000881 | 6.937689 | 1.71E-15 |
| 86 |  | cg01028142 | -0.2434 | 1.28E-05 | 6.937689 | 1.71E-15 |
| 87 | EPSTI1 | cg26422453 | -0.13386 | 0.001008 | 3.555424 | 4.75E-08 |
| 88 |  | cg12439472 | -0.29726 | 0.000014 | 3.555424 | 4.75E-08 |
| 89 |  | cg03763873 | -0.1049 | 1.76E-05 | 3.555424 | 4.75E-08 |
| 90 | IRF7 | cg08926253 | -0.10387 | 2.24E-05 | 3.410812 | 8.04E-08 |
| 91 | USP18 | cg14293575 | -0.28342 | 2.24E-05 | 6.389357 | 1.33E-13 |
| 92 | IFIT1 | cg05552874 | -0.26645 | 0.000028 | 8.496348 | 2.94E-17 |
| 93 | LY6E | cg12110437 | -0.16041 | 0.000115 | 3.395492 | 1.40E-07 |
| 94 |  | cg14392283 | -0.31757 | 4.51E-05 | 3.395492 | 1.40E-07 |
| 95 | IFIH1 | cg08888522 | -0.12573 | 6.26E-05 | 2.029833 | 2.29E-03 |
| 96 | HERC5 | cg09053843 | -0.12368 | 0.000496 | 4.16163 | 9.72E-10 |
| 97 |  | cg11029522 | -0.10385 | 7.93E-05 | 4.16163 | 9.72E-10 |
| 98 | SPATS2L | cg13144059 | -0.13234 | 3.94E-05 | 4.507602 | 9.48E-10 |
| 99 |  | cg04944232 | -0.12147 | 0.000139 | 4.507602 | 9.48E-10 |
| 100 |  | cg03764410 | -0.12097 | 8.64E-05 | 4.507602 | 9.48E-10 |
| 101 | EIF2AK2 | cg16795804 | -0.10673 | 0.000485 | 3.093871 | 8.97E-07 |
| 102 |  | cg14126601 | -0.17385 | 0.000199 | 3.093871 | 8.97E-07 |
| 103 | IFIT5 | cg05729683 | -0.17228 | 0.000453 | 2.53198 | 9.66E-05 |
| 104 | IFIT3 | cg06188083 | -0.14485 | 0.000553 | 10.8863 | 4.19E-22 |
| 105 | BCL2 | cg11681041 | -0.12291 | 0.000602 | 1.588692 | 4.01E-02 |
| 106 | RSAD2 | cg15839328 | -0.16738 | 0.00113 | 6.926518 | 5.67E-14 |
| 107 |  | cg10771443 | -0.15901 | 0.000699 | 6.926518 | 5.67E-14 |
| 108 | IFI44 | cg01079652 | -0.17402 | 0.000767 | 4.526514 | 1.14E-10 |
| 109 | STAT1 | cg00676801 | -0.11986 | 0.004558 | 2.056474 | 1.48E-03 |
| 110 |  | cg14951497 | -0.15017 | 0.000879 | 2.056474 | 1.48E-03 |
| 111 | TCF7L2 | cg22795218 | -0.15737 | 0.001419 | 1.803167 | 5.17E-03 |
| 112 | IFIT2 | cg23928123 | -0.10781 | 0.003364 | 3.532472 | 2.22E-08 |
| 113 | HERC6 | cg02521291 | -0.12336 | 0.004662 | 3.158465 | 1.04E-06 |
| 114 | BICC1 | cg12342675 | -0.2515 | 0.005288 | 6.570242 | 3.24E-03 |
| 115 | DRAM1 | cg27425327 | -0.11882 | 0.00768 | 1.97093 | 1.74E-03 |
| 116 | LTBP1 | cg14260773 | -0.23074 | 0.018103 | 2.107883 | 6.76E-03 |
| 117 |  | cg18213786 | -0.11429 | 0.009508 | 2.107883 | 6.76E-03 |
| 118 | DDX60L | cg15735316 | -0.10853 | 0.011613 | 1.763527 | 1.20E-02 |
| 119 | ISG15 | cg20062691 | -0.10514 | 0.011803 | 5.771903 | 2.33E-13 |
| 120 | ANKRD33B | cg17271308 | -0.328 | 0.012556 | 1.704699 | 2.81E-02 |
| 121 | NFASC | cg07475752 | -0.3737 | 0.013931 | 3.49559 | 1.49E-03 |
| 122 | PTGFR | cg27046936 | -0.11474 | 0.014081 | 34.96612 | 2.62E-07 |
| 123 | ABCA13 | cg15579246 | -0.22278 | 0.017019 | 3.222749 | 2.69E-04 |
| 124 | LAP3 | cg02294690 | -0.11216 | 0.028102 | 3.181603 | 6.18E-07 |
| 125 | GALNT13 | cg24232932 | -0.10522 | 0.036421 | 2.943756 | 1.62E-02 |
| 126 | CLEC1B | cg18037388 | -0.31907 | 0.043064 | 1.550804 | 4.07E-02 |
| **Hypomethylation+downregulation** | | | | | | |
| 127 | ESPNP | cg24875017 | -0.10837 | 0.005875 | 0.418872 | 1.70E-03 |
| 128 | DCHS2 | cg15926738 | -0.37234 | 0.018151 | 0.105004 | 6.44E-09 |
| 129 | ST5 | cg17046776 | -0.13913 | 0.024668 | 0.322815 | 7.58E-06 |

| **S-Table 7. Adjust analysis for the three DMPs** | | | |
| --- | --- | --- | --- |
| **Factor** | **OR** | **95% CI** | ***P*-value** |
| cg08332381 | | | |
| cg08332381 | 592.571 | 2.502-140318.734 | 0.022 |
| Age(year) | 1.028 | 0.909-1.162 | 0.663 |
| SLEDAI | 1.09 | 0.761-1.561 | 0.639 |
| Hydroxychloroquine sulfate | 2.807 | 0.018-65.461 | 0.521 |
| Immunosuppressant | 0.204 | 0.018-2.343 | 0.202 |
| Glucocorticoid | 3.025 | 0.024-388.296 | 0.655 |
| cg03297029 | | | |
| cg03297029 | 575244.8 | 2.869-1.154E+11 | 0.033 |
| Age(year) | 1.059 | 0.905-1.239 | 0.476 |
| SLEDAI | 1.583 | 0.933-2.686 | 0.089 |
| Hydroxychloroquine sulfate | 2.178 | 0.054-87.391 | 0.679 |
| Immunosuppressant | 3.727 | 0.083-167.024 | 0.498 |
| Glucocorticoid | 10.98 | 0.160-753.260 | 0.267 |
| cg16797344 | | | |
| cg16797344 | 0.003 | 0-0.362 | 0.018 |
| Age(year) | 1.01 | 0.881-1.157 | 0.89 |
| SLEDAI | 1.34 | 0.92-1.952 | 0.127 |
| Hydroxychloroquine sulfate | 0.669 | 0.024-18.745 | 0.669 |
| Immunosuppressant | 0.811 | 0.054-12.218 | 0.811 |
| Glucocorticoid | 9.38 | 0.242-363.039 | 9.38 |
| OR, odds ratio; CI, confidence interval; SLEDAI, systemic lupus erythematosus disease activity index | | | |

| **Supplementary Table 8. Primers and detailed information for validated genes in vivo experiments** | | | | | |
| --- | --- | --- | --- | --- | --- |
| Gene | DMP | location | Pyrosequencing | | Premiers for RT-qPCR |
|  |  |  | Outer primers | Inner primers |  |
| IFI27 | cg10778971 | 5'UTR-opensea | - | F: GGGGTGGAGTTGTTTAAAAGG | F: ACAGTTGTGATTGGAGGAGTTGTG |
|  |  |  |  | R: CCACAACCCACCCAAAAT | R: GCAATGGCAGACCCAATGGA |
| TNFRSF9 | cg08840010 | 5'UTR-shelf | F: AGTTGAGGATTAGTAATAGAGTGGTT | F: AGAGTGGTTATTATGTTGTAATAGTTG | F: GTCTGTGGACCATCTCCAGC |
|  |  |  | R: AATAAAAAATACACCCTCAAACTTTAACA | R: AATAAAAAATACACCCTCAAACTTTAACA | R: TGTACTGGTCTCATAAATGGTTGTT |
| BCL2L14 | cg20481287 | 5'UTR-opensea | - | F: GGGAGGTATGTAATGATATGATAGT | F: AGGAAGATTCGCAGAGCACG |
|  |  |  |  | R: ATCTCAAACCAAAAATTCCCATACT | R: CTTTGGGGTCCACAGCTTCAT |
