# Supplementary figures and images for: DNA methylation and whole-genome transcription analysis in CD4+ T cells from systemic lupus erythematosus patients with or without renal damage
Source: Clin Epigenetics. 2024 Jul 30;16:98. doi: 10.1186/s13148-024-01699-7 (PMC11290231; doi:10.1186/s13148-024-01699-7)

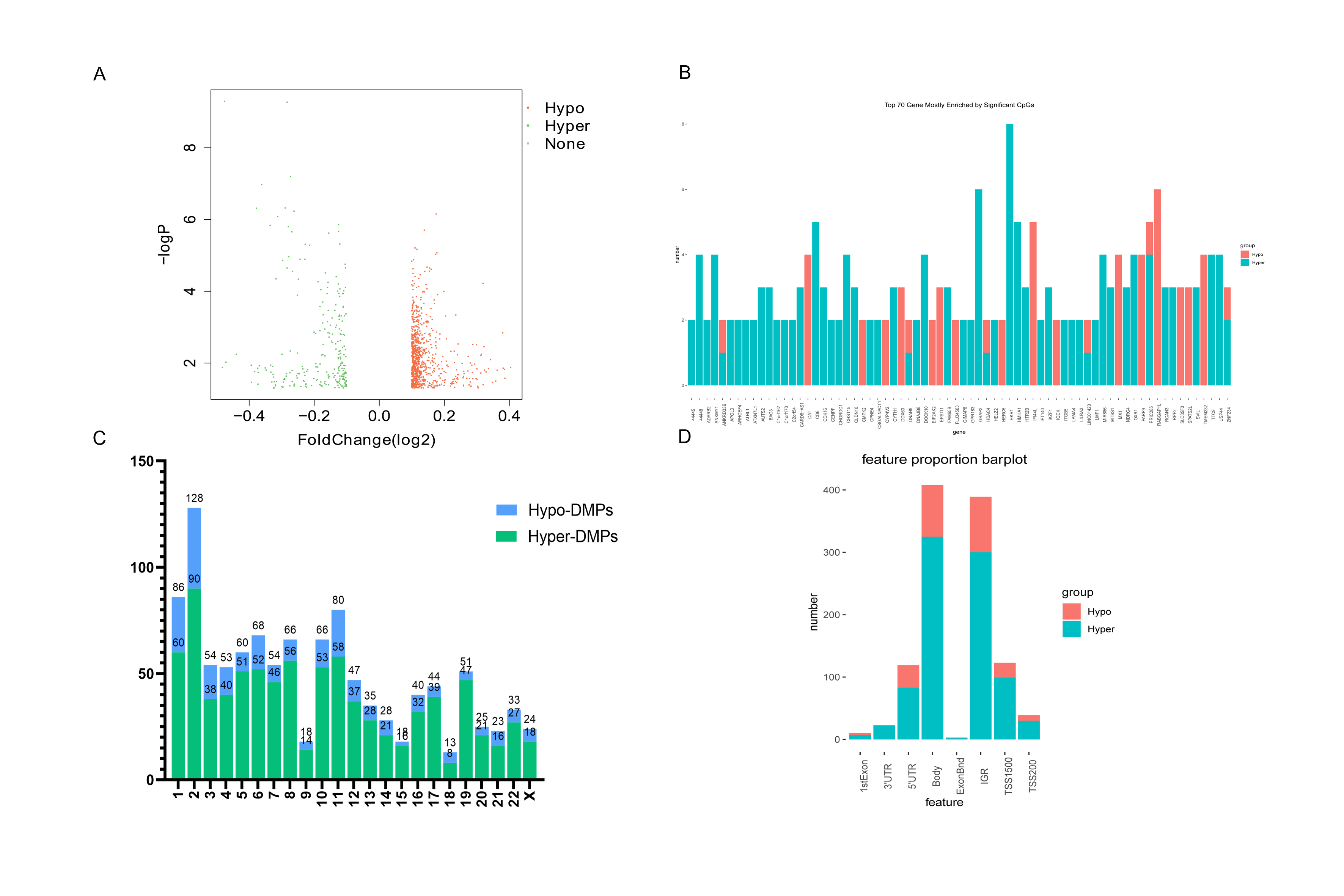

Supplement: Supplementary file 2 — Additional file 2: Figure 1. DMP between SLE-NKI group and the healthy control group. A: The volcano plot for DMP; B: Top 70 genes mostly enriched by significant CpGs. Taking the gene name as the X-axis, the number of DMP as the Y-axis. C: The distribution of DMP among chromosomes. D: The distribution of DMP in the gene. [file 13148_2024_1699_MOESM2_ESM.tif]

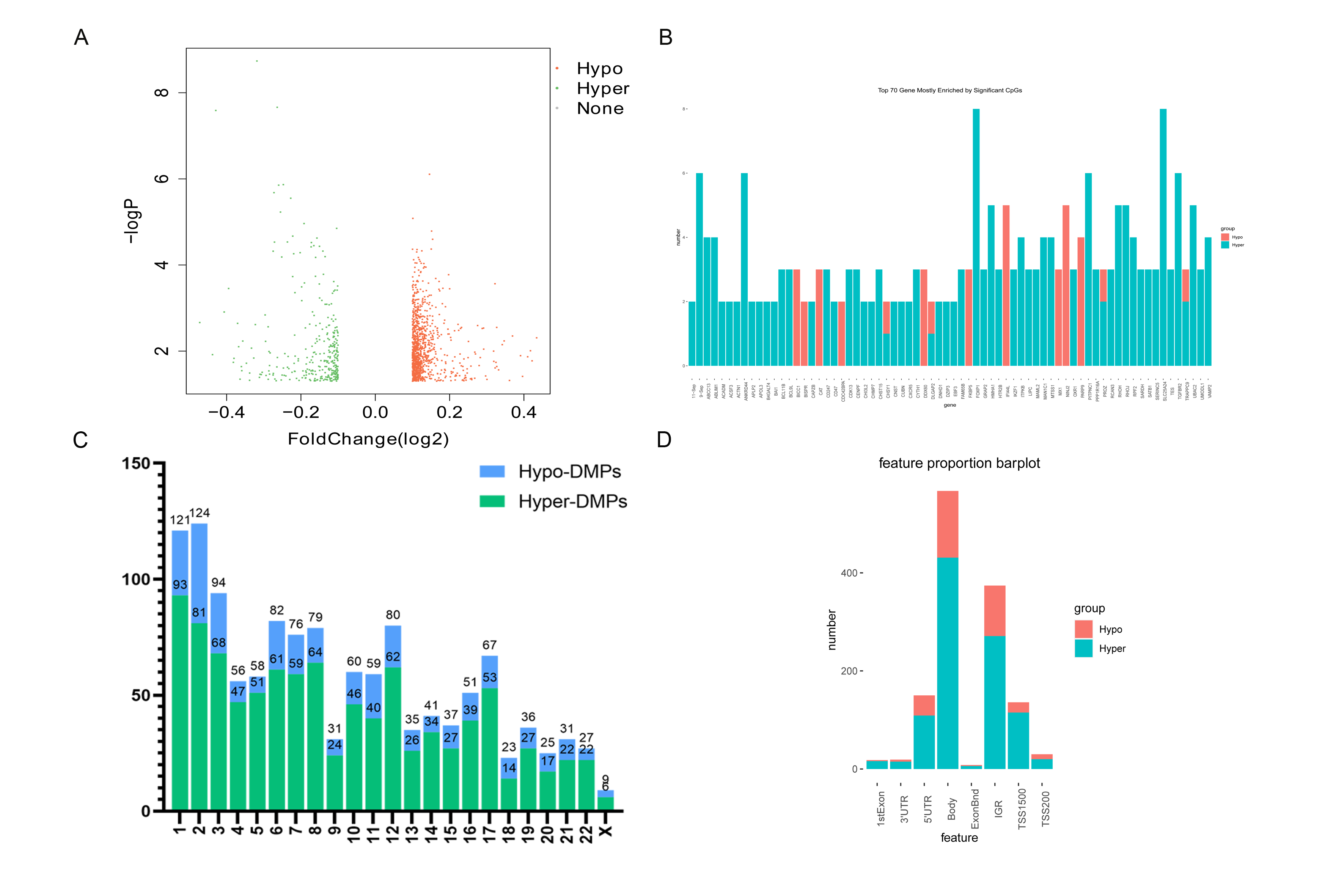

Supplement: Supplementary file 3 — Additional file 3: Figure 2. DMP between LN group and healthy control group. A: The volcano plot for DMP; B: Top 70 genes mostly enriched by significant CpGs. Taking the gene name as the X-axis, the number of DMP as the Y-axis. C: The distribution of DMP among chromosomes. D: The distribution of DMP in the gene. [file 13148_2024_1699_MOESM3_ESM.tif]

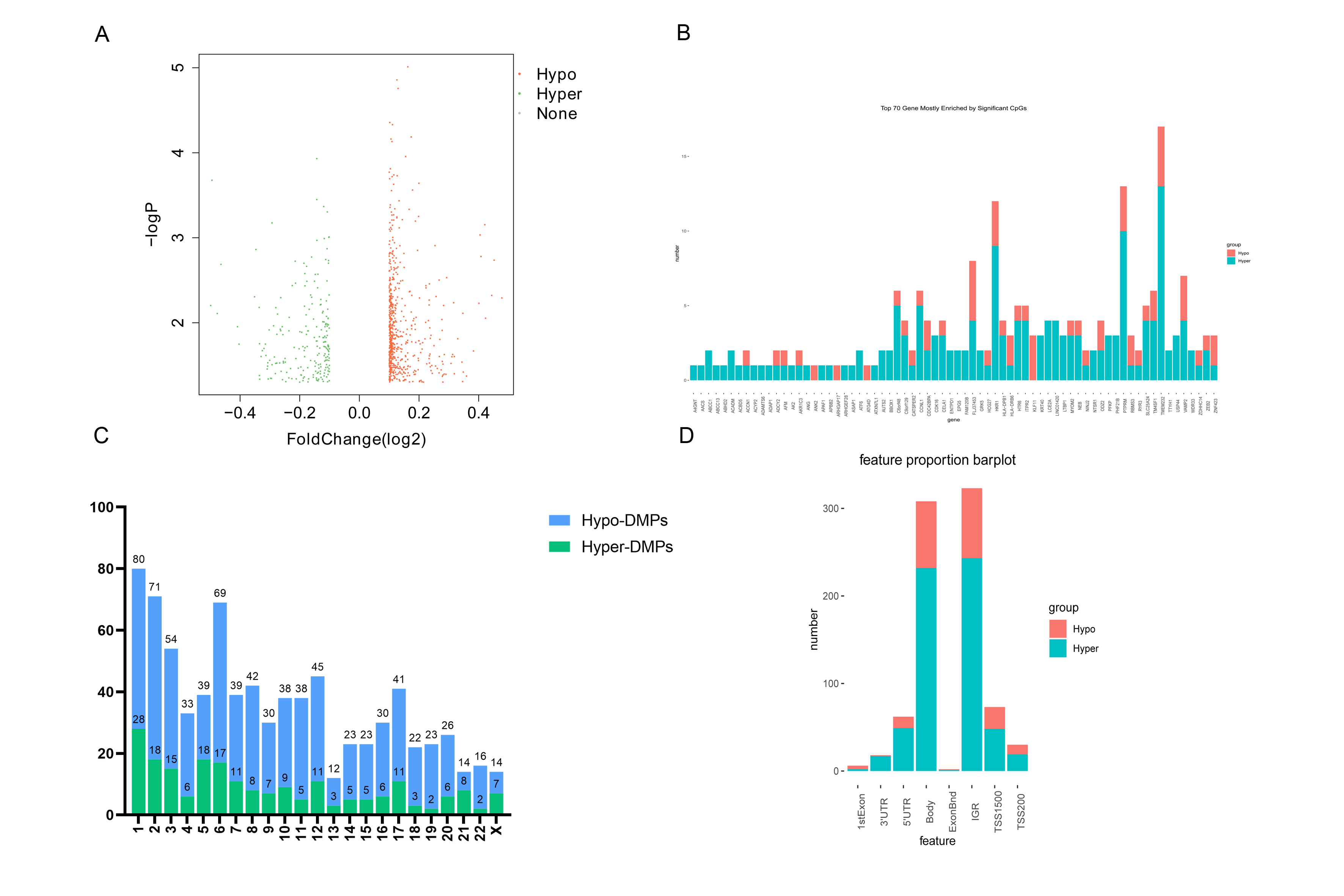

Supplement: Supplementary file 4 — Additional file 4: Figure 3. DMP between LN group and SLE-NKI group. A: The volcano plot for DMP; B: Top 70 genes mostly enriched by significant CpGs. Taking the gene name as the X-axis, the number of DMP as the Y-axis. C: The distribution of DMP among chromosomes. D: The distribution of DMP in the gene. [file 13148_2024_1699_MOESM4_ESM.tif]

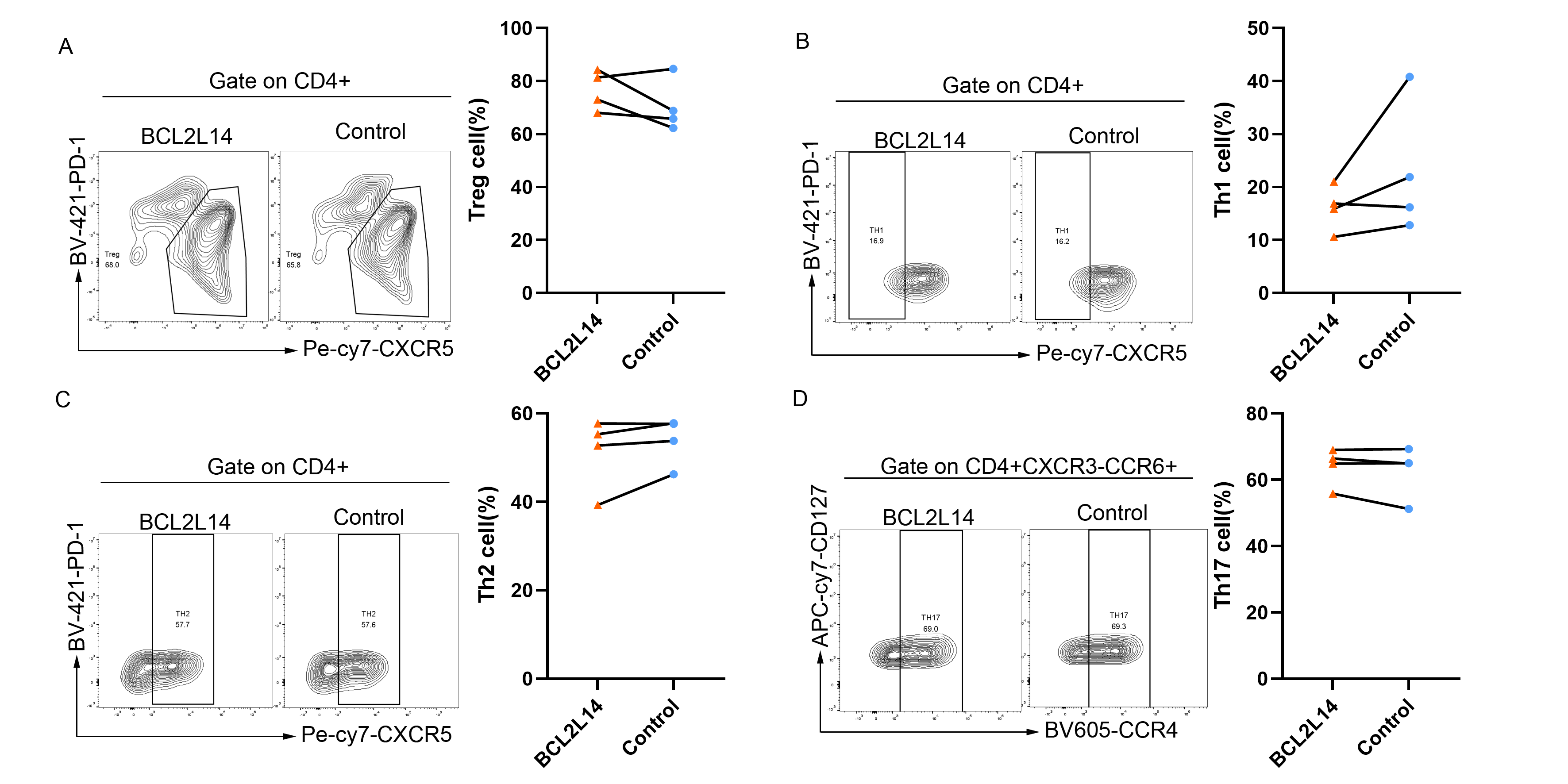

Supplement: Supplementary file 5 — Additional file 5: Figure 5. FACS analysis of differentiation ratio of Treg, Th1, Th2, and Th17 cells in CD4+T cells with BCL2L14 overexpression, which all have no differences. A: Treg cells; B: Th1 cells; C: Th2 cells; D:Th17 cells. [file 13148_2024_1699_MOESM5_ESM.tif]
